# Biochemical evidence that the whole compartment activity behavior of GAPDH differs between the cytoplasm and nucleus

**DOI:** 10.1371/journal.pone.0290892

**Published:** 2023-08-31

**Authors:** Helen S. Tang, Chelsea R. Gates, Michael C. Schultz

**Affiliations:** Department of Biochemistry, University of Alberta, Edmonton, Alberta, Canada; Midwestern University, UNITED STATES

## Abstract

Some metabolic enzymes normally occur in the nucleus and cytoplasm. These compartments differ in molecular composition. Since post-translational modification and interaction with allosteric effectors can tune enzyme activity, it follows that the behavior of an enzyme as a catalyst may differ between the cytoplasm and nucleus. We explored this possibility for the glycolytic enzyme glyceraldehyde 3-phosphate dehydrogenase (GAPDH). Homogenates of pristine nuclei and cytoplasms isolated from *Xenopus laevis* oocytes were used for whole compartment activity profiling in a near-physiological buffer. Titrations of NAD^+^ revealed similar whole compartment activity profiles for GAPDH in nuclear and cytoplasmic homogenates. Surprisingly however GAPDH in these compartments did not have the same behavior in assays of the dependence of initial velocity (*v*_0_) on G3P concentration. First, the peak *v*_0_ for nuclear GAPDH was up to 2.5-fold higher than the peak for cytoplasmic GAPDH. Second, while Michaelis Menten-like behavior was observed in all assays of cytoplasm, the *v*_0_ versus [G3P] plots for nuclear GAPDH typically exhibited a non-Michaelis Menten (sigmoidal) profile. Apparent K_m_ and V_max_ (G3P) values for nuclear GAPDH activity were highly variable, even between replicates of the same sample. Possible sources of this variability include *in vitro* processing of a metabolite that allosterically regulates GAPDH, turnover of a post-translational modification of the enzyme, and fluctuation of the state of interaction of GAPDH with other proteins. Collectively these findings are consistent with the hypothesis that the environment of the nucleus is distinct from the environment of the cytoplasm with regard to GAPDH activity and its modulation. This finding warrants further comparison of the regulation of nuclear and cytoplasmic GAPDH, as well as whole compartment activity profiling of other enzymes of metabolism with cytosolic and nuclear pools.

## Introduction

During the first 75 years of the 20^th^ century, research on the cell biology of the nucleus included an intense effort to determine if this cell compartment houses classical metabolic enzymes [1–3]. This work exploited well established histochemical and biochemical methods for enzyme activity detection. Based on these studies, many investigators endorsed the notion that in some cell types, some metabolic enzymes have a pool in the nucleus as well as the cytoplasm. Among them was Vincent Allfrey of Rockefeller University (New York, U.S.A.). In a 1975 discussion concerning the enzyme activity profile of purified nuclei, Allfrey stated that “… the lymphocyte nucleus has a complete glycolytic cycle and many components of the citric acid cycle…” [4].

The work of cell biologists during these pioneering years extended beyond qualitative assessment of enzyme expression in the nucleus. One thrust was to quantitate enzyme activity to determine if it can differ between the cytoplasm and nucleus. For example, Kato and Lowry (1973) microdissected slices of cytoplasm and nucleus from frozen tissue sections and measured activity by enzymatic cycling in reaction volumes as small as 1 nL [5]. In this study, five of seven enzymes tested were reported to have higher specific activity in the nucleus than the cytoplasm (with the caveat that measures of total compartment protein were used as a proxy for enzyme amount). A promising method for enzyme/compartment comparison by detailed kinetic analysis was introduced. Profiles of activity dependence on substrate concentration were generated for nuclear and cytoplasmic extracts at different temperatures and pH. These profiles were found to differ, possibly because distinct enzyme “types” are expressed in the cytoplasm and nucleus [1].

Despite early enthusiasm for the idea that the nuclear and cytoplasmic pools of enzymes could have different properties as catalysts, research in this field declined precipitously after the 1970s. This decline preceded the current resurgence of interest in metabolic regulation, and ironically, the discovery of new molecular mechanisms that could contribute to differential enzyme regulation according to cell location. For example, we now know that the nuclear accumulation of a metabolic enzyme can depend on its binding to a protein inhibitor of catalytic activity (nuclear glucokinase inhibition by glucokinase regulatory protein) [6]. Furthermore, it is now clear that the catalytic activity of some nucleocytosolic enzymes is controlled by post-translational modification. For example, the activity of acetyl-CoA synthetase 2 (ACSS2/AceCS1) [7] is controlled by reversible lysine acetylation [8]. One ACSS2 deacetylase is known to shuttle between the cytoplasm and nucleus [9], raising the possibility that the nuclear and cytoplasmic pools of ACSS2 can have different levels of acetylation and therefore activity. Finally, differential regulation of the nuclear and cytosolic pools of an enzyme could result from differential accumulation of allosteric metabolites. This possibility flies in the face of the long-held view that metabolites rapidly equilibrate between the cytoplasm and nucleus. Now however, it seems likely that at least one critical cellular metabolite (NAD^+^) can have a different concentration in the nucleus and cytosol [10].

Encouraged by this new knowledge pertaining to enzyme regulation, we sought evidence consistent with the hypothesis that the activity behavior of a glycolytic enzyme can differ between the cytoplasm and nucleus. The enzyme is glyceraldehyde 3-phosphate dehydrogenase (GAPDH). It catalyzes the reaction glyceraldehyde 3-phosphate + NAD^+^ + P_i_ ↔ 1,3 bisphosphoglycerate + NADH + H^+^ (in short, G3P + NAD^+^ ↔ BPG + NADH). The following facts supported a focus on GAPDH. It has long been known to exist in the cytoplasm and nucleus and contribute to glycolysis in both compartments [11,12]. Its biochemical characterization has been reported for more than 200 species including the animal model used here (*X*. *laevis* [13], BRENDA:EC1.2.1.12 in Brenda Enzyme Database). And the available kinetic data is complemented by knowledge of the three-dimensional structure of GAPDH of several mammalian species including human [14–17].

GAPDH further attracted our attention as a focus for analysis of functional partitioning by cell location because of what is known about its physiological regulation. It is a promising drug target for the treatment of cancer and parasitic diseases, and some autoimmune conditions are responsive to an inhibitor of its activity [17]. It is known to associate with a multitude of proteins, some of which are expressed in the cytoplasm and nucleus [16]. Its activity is regulated by association with a glutathione S-transferase [18], Ca^2+^-dependent phosphorylation [19], lysine acetylation [20], succination [21], redox state [22,23], arginine methylation [24], non-enzymatic or perhaps autocatalytic malonylation and palmitoylation [25,26] and malonylation-dependent binding to RNA [27]. The observation that interactions with 8 metabolites can cause conformational changes in the E. coli enzyme [28] suggests a much broader potential for allosteric regulation than previously appreciated. Finally, there has been no detailed comparison of GAPDH activity in the nucleus and whole cytoplasm of any cell type.

**Fig 1:**
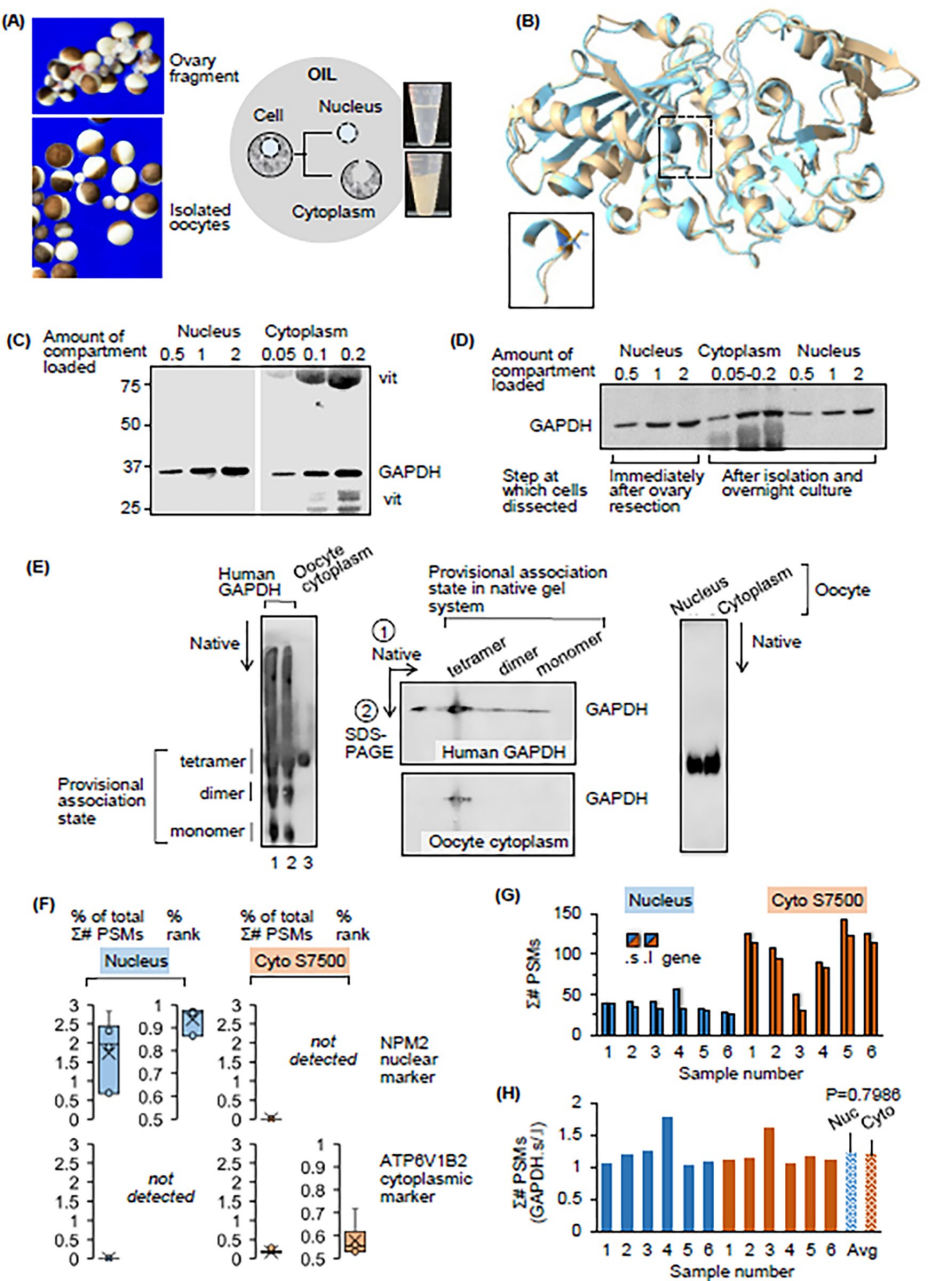
GAPDH protein in the oocyte nucleus and cytoplasm. (A) Oocyte isolation. Oocytes are freed from ovary fragments (top left photo) by collagenase digestion. Mechanical treatment yields isolated oocytes without adhering somatic cells (bottom photo). Cells are dissected under oil to obtain nuclei and cytoplasms, which are then homogenized in a physiological buffer (right panel). (B) GAPDH structure. Superimposition of the structure of the human GAPDH monomer (gold) and a model of *X*. *laevis* GAPDH generated by RoseTTAFold (cyan). The pullout shows the catalytic cysteine (C152 and 150 in human and frog respectively). (C) GAPDH protein in oocyte nucleus and cytoplasm. Western blotting analysis of GAPDH in ensemble preparations of nuclear and cytoplasmic homogenate from isolated oocytes. vit, full length and processing products of vitellogenin, a storage protein that accumulates exclusively in cytoplasmic yolk granules. (D) Nuclear GAPDH expression: Effect of oocyte isolation and culture. Western blotting analysis of GAPDH in nuclei dissected directly from oocytes in ovary fragments (left three lanes) and in fractions obtained from isolated oocytes (right six lanes). (E) Self-association state of GAPDH in the nucleus and cytoplasm. Analysis of GAPDH by native PAGE (left-most and right-most panels), and native PAGE followed by conventional SDS-PAGE (middle panel). Recombinant human GAPDH and oocyte GAPDH were detected by Western blotting. The three fastest migrating species of human GAPDH resolved by native PAGE are provisionally identified as the enzyme’s monomeric, dimeric and tetrameric forms. GAPDH in the oocyte cytoplasm comigrates with human tetrameric GAPDH (left panel) and oocyte nuclear GAPDH (right panel). (F)–(H) Validation of whole compartment LC-MS analysis and LC-MS profiling of GAPDH.s and GAPDH.l by compartment. (F) Relative abundance of marker proteins in the oocyte nucleus and yolk-free cytoplasm. Box and whisker plots of protein expression in six individual stage VI nuclei (blue bars) and the 7,500 x g supernatant obtained from six individual stage VI cytoplasms (cyto S7500, orange bars). % of total Σ# peptide spectral matches (PSMs) is an estimate of relative protein abundance. X is the mean. Abundance rankings (% rank in each sample) are shown in the right hand plots. The histone chaperone NPM2 was present in all nuclei, but not detectable in the cytoplasms. The cytoplasmic marker ATP6V1B2 (a subunit of the vacuolar H^+^-ATPase) was only detected in cytoplasm. This validation result applies to the analysis in Figs 1G, 1H and 3A. (G) Raw number of PSMs assigned to GAPDH.s and GAPDH.l (bars with dotted and solid outlines respectively) in the six nuclear homogenates and six cytoplasmic S7500s analyzed. (H) For each sample, ratio of Σ# PSMs assigned to GAPDH.s versus GAPDH.l. The last two bars summarize the data (errors are SD, P = 0.796773).

Three facts uniquely suit our model of choice—the *X*. *laevis* oocyte–to analysis of GAPDH catalytic behaviour by cell compartment. First, its cytoplasm and nucleus can be obtained in near-native state, without loss or mixing of contents (Fig 1A) [29]. This is done by manual dissection of cells under oil, which is straightforward because of the oocyte’s large size (full grown diameter of 1.2–1.3 mm) [30]. Second, the absolute nuclear and cytosolic concentrations of oocyte GAPDH have been determined by quantitative mass spectrometry [31] (S1 Fig) and corroborated by data in an independent study [32]. Third, based on the quantitative mass spectrometry data and the known amount of cytoplasmic material that is recovered with oil-isolated nuclei, we estimate that GAPDH in the nucleoplasm (nucleosol and nuclear structures it suspends) is in approximately 1,000-fold excess of any GAPDH that might be in cytoplasm associated with isolated nuclei (S2 Fig). Therefore, the readout of assays of GAPDH activity associated with isolated nuclei will be predominated by the signal from enzyme located in the nucleoplasm.

We have leveraged these advantages to compare the enzymatic behavior of the pools of nuclear and cytoplasmic GAPDH. Activity of cytoplasmic and nuclear GAPDH was assayed in reactions containing the entire contents of the cytoplasm and nucleus respectively. Compartment samples were diluted in a physiological buffer and reaction progress at varying concentrations of G3P and NAD^+^ was assessed by non-invasive spectrophotometric detection of the reaction product NADH. This whole compartment approach was not pursued in order to obtain estimates of kinetic parameters from which formal mechanistic models of GAPDH activity could be derived (this goal is best achieved by analysis of highly pure enzyme). Rather, in assaying enzyme behavior in whole compartment homogenates in a physiological buffer, we hoped to capture biochemical features of activity regulation that would be disrupted or lost upon enzyme purification. Indeed, analysis of the initial velocity (*v*_0_) data has revealed unexpected differences of GAPDH enzymatic behavior in whole homogenates of the nucleus and cytoplasm.

## Materials and methods

### Oocyte isolation and maintenance

Adult female *X*. *laevis* (Xenopus One, MI, U.S.A.) were anaesthetized using tricaine methanesulfonate and euthanized by decapitation according to procedures approved by the Health Sciences Animal Care and Use Committee of the University of Alberta (Animal Use Protocol # 00000942). All animals were euthanized at the same time of day (11:00–11:30). Ovaries were removed to OR-2 medium (5 mM Hepes-NaOH pH 7.8, 82.5 mM NaCl, 2.5 mM KCl, 1 mM MgCl_2_, 1 mM CaCl_2_, 1 mM Na_2_HPO_4_) [33] and cut up with scissors to obtain tissue pieces that contain approximately 30 full-grown oocytes. These were washed in OR-2 and digested by collagenase treatment at room temperature to obtain defolliculated oocytes. The volume of the ovary fragments was estimated using a graduated cylinder, for digestion at 1.7 mL of collagenase solution per approximately 1 mL of tissue. The collagenase solution was 3 mg/mL collagenase and 1 mg/mL bovine serum albumin (BSA) in OR-2 [33]. Digestion was performed in 15 or 50 mL Falcon tubes that were approximately ¾ full of tissue pieces in collagenase solution. The tubes were rotated at 20 rpm using a Fisherbrand^TM^ Mini Tube Rotator (# 88861051). Small samples were taken during digestion to monitor the release of oocytes from their follicles. Suitable populations of defolliculated oocytes were obtained after 1.25 to 2 hr of collagenase digestion. These oocytes remain associated with various somatic cells [34,35]. They were freed of such cells by a bulk mechanical treatment, specifically gentle rocking on abrasive paper (modified from reference 35). This procedure was performed in plastic Petri dishes with a well-fitting disc of unused waterproof silicon carbide abrasive paper (sia Abrasives Industries AG, # 1727 siawat P1200) attached to the dish base using Double-Coated Scotch® tape (3M # 667C). After it was attached to the plastic, and immediately before use, the abrasive paper was briefly washed with OR-2. The configuration that ensured free movement of stage V/VI oocytes in a single layer was to process a settled volume of 2.8 mL of collagenased cells in a 100 x 15 mm abrasive-lined dish also containing 12.5 mL of OR-2. The dish lid was sealed in place using Parafilm® M for 3D agitation (nutating motion) at a setting sufficient for cell rolling but not splashing of the suspension or trapping of cells at the junction of the lid and base. Detachment of somatic cells was monitored by DAPI staining (49,6-diamidino- 2-phenylindole, 1 μg/mL) [36] of randomly selected oocytes. Complete loss of somatic cells was observed after 15–20 min of debriding. Isolated oocytes were maintained in BioLite^TM^ tissue culture dishes (Thermo Scientific^TM^ # 130182) in OR-2 with penicillin and streptomycin (100 μg/mL each) and used within 3 d of isolation.

### Dissection of isolated oocytes and oocytes in ovary fragments

Isolated oocytes were dissected to obtain near-native cytoplasms and nuclei. Wicking of aqueous medium from oocytes and manual dissection under mineral oil were performed according to Paine et al. [29]. Some nuclei were dissected not from isolated oocytes, but from oocytes that were in ovary fragments. The latter dissections were performed immediately after mincing of the ovary, as follows. Ovary pieces were immobilized on Whatman Grade 3MM paper to remove the OR-2, then immersed in mineral oil. Direct cell dissection involved making a single scissor cut into an ovary fragment, through the thecal tissue surrounding an oocyte and into its animal pole. Vannas spring scissors with a 4 mm cutting edge (Fine Scientific Tools # 15018–10) were ideal for this purpose. Slicing of the oocyte was followed by nuclear removal using 18G needles.

For large-scale homogenates, nuclei and cytoplasms obtained by dissection at room temperature were pooled under mineral oil at 6°C in a Nunc 35 mm Petri dish (ThermoFisher Scientific # 150318) viewed with a dissecting microscope (see section “The activity behavior of nuclear and cytoplasmic GAPDH”). Cooling of the dish was achieved using a Physitemp TS4-SMP small Petri dish thermal microscope stage with external temperature monitoring using a Qualitrol® Nomad fiber optic thermometer and a Neoptix fiber optic probe (T1 sensor T1C-2M-PP10, 0.6 mm tip). Cooling the oil to 4°C was not effective because ice formed on the surface of the oil.

### Structural model of *X*. *laevis* GAPDH

The model was based on the *X*. *laevis* sequence for gapdh.s in Xenbase [37] (Xenbase XB-GENEPAGE-487819, UniProt G3P_XENLA). It is model 1 generated by RoseTTAFold [38], with predicted I-DDT (Distance Difference Test) score using DeepAccNet = 0.92. The overlay of this model with human GAPDH (1u8f 0 chain.pdb) was generated using ChimeraX 1.3 [39].

### Preparation of cytoplasmic and nuclear homogenates

Cell compartments were dispersed on ice in “Homogenization and Reaction” (HR) buffer. HR buffer is “Isolation Medium with Mg^2+^” of Gall and colleagues [40,41] (IM) supplemented with dithiothreitol and glycerol (83.0 mM KCl, 17.0 mM NaCl, 6.5 mM Na_2_HPO_4_, 3.5 mM KH_2_PO_4_, 1.0 mM MgCl_2_, 1 mM DTT, 20% glycerol, pH 7.4). Homogenization was achieved by trituration (5 pipettings at the volume of buffer used for homogenization) and 5 sec vortexing at 7500 rpm. Nuclear homogenate was frozen at 1 nucleus/20 μL HR buffer. The preparation of cytoplasmic homogenate for freezing was scaled to approximate the approach used for nuclei, taking into account the typical values of 2.5 μg protein/nucleus and 25 μg of cytosolic protein/stage VI oocyte [42]. Specifically, cytoplasms were homogenized in 125 μL of HR buffer per one cytoplasm; 12.5 μL of such homogenates contain approximately the same amount of cytosolic protein as there is total protein in a stage VI oocyte nucleus.

### GAPDH enzyme activity measurement

Preparation of stock reagents. 100 mM pyrophosphate (PP_i_) and 100 mM NAD^+^ were prepared by dissolving sodium pyrophosphate decahydrate (MP Biomedicals # 152579) and β-nicotinamide mononucleotide (Sigma-Aldrich # N3501) in IM. 100 mM G3P was prepared by diluting DL-glyceraldehyde 3-phosphate solution (Sigma Aldrich # G5251) in IM. PP_i_, NAD^+^, and G3P were stored at -20°C in small aliquots until use. 100 mM arsenate was prepared fresh for each assay by dissolving sodium arsenate dibasic heptahydrate in IM.

Assay method. Assays were performed in Corning® 384 Well Deep Well Plates (Sigma # CLS3347) and absorbance data was collected using a plate reader (BioTek Synergy 4 with Hybrid Technology^TM^). Whole reactions were prepared by mixing a “varying substrate” cocktail with a “constant substrate” cocktail. The “varying substrate” mixture consisted of the GAPDH sample and 0 to 5 mM of the varying reagent (depending on the experiment) for titration; this makes up ¼ of the final reaction volume and contained 1 mM DTT and 20% glycerol. The varying substrate cocktails were premixed by 7 sec of vortexing and hand pipetted into wells in triplicates. The “constant substrate” mix contained G3P or NAD^+^ as indicated in the figures and the additional constant reagents (10 mM arsenate and 5 mM PP_i_). The constant substrate mix was dispensed into the wells by the plate reader at 300 μl/sec to facilitate mixing. After dispensing, the final volume in each well was 40 μL. The final makeup of the whole reaction in each well was 5 mM PP_i_, 10 mM arsenate, and 5% glycerol (all other components were at the concentrations of HR buffer). Absorbance at 340 nm was read every 30 sec using the kinetic protocol of the Biotek “Gen 5 Microplate Reader and Imaging software”. A_340_ measurements from the initial three minutes were used to calculate initial rates. The assay blanks contained purified GAPDH or oocyte homogenate but no substrates. For the experiments in Figs 6 and 7 the amount of GAPDH monomer in each assay is 0.0388 picomoles. Data were analyzed using Microsoft Excel and GraphPad Prism.

### SDS-PAGE, native PAGE, 2D PAGE (native + SDS-PAGE); Western blotting

#### Homogenate preparation

Nuclei were collected in groups of 20 and dispersed in 10 μL of HR buffer by ten mixings in a 10 μL micropipette tip. The homogenates were then brought to a final concentration of 1 nucleus/μL with HR buffer, and further mixed by vortexing for 10 sec. Cytoplasms were collected in groups of 10 and homogenized in HR buffer to a final concentration of 0.1 cytoplasm/μL. The homogenates were stored in 20 μL aliquots at -80°C until use.

#### SDS-PAGE and Western blotting

Qualitative analysis was performed by two methods which yielded similar results. The conventional method used SDS-12% polyacrylamide gels and a submarine transfer system for blotting to nitrocellulose [43]. The updated method used the Bio-Rad Trans-Blot Turbo Transfer System for blotting (Bio-Rad #s 1704150, 1704271). Membranes were blocked in 4% BSA in TBST (20 mM Tris base pH 8.2, 150 mM NaCl, 0.1% Tween 20) for 1 hr with gentle agitation and washed with TBST (5 x 5 min). GAPDH was detected using mouse monoclonal antibody D-6 (Santa Cruz Biotechnology # sc-166545, overnight incubation at 1:5000 with 4% BSA). TBST washing as above was followed by incubation for 1 hr at room temperature in 1:10000 goat anti-mouse IgG (H+L)-HRP conjugate (Bio-Rad # 1706516) in 4% BSA. The membrane was next washed in TBST and incubated in the Amersham™ ECL™ Western Blotting Detection Reagents at a 1:1 ratio. The chemiluminescent signals were captured by exposure to film (GE Healthcare, Amersham Hyperfilm^TM^ ELC) or by use of a LI-COR digital imager (Odyssey® XF Imaging System). Quantitative analysis was performed according to the updated method outlined above, except that the gel was equilibrated in transfer buffer with SDS (25 mM Tris, 190 mM glycine, 20% methanol, 0.05% SDS) before electroblotting. Integrated band intensities were obtained using Image Studio Lite Quantification Software v.5.2 (LI-COR Biosciences).

#### Native PAGE and Western blotting

Invitrogen 4–16% NativePAGE^TM^ Bis-Tris Mini Protein Gels (10 wells), NativePAGE Running Buffer and NativePAGE Cathode Buffer Additive were used for native gel electrophoresis (ThermoFisher Scientific #s BN1002BOX, BN2001 and BN2002 respectively). Gels were loaded with up to 0.5 nuclei, 0.05 cytoplasm, and 1 μg of ProSpec human recombinant GAPDH (# ENZ-350) in sample buffer from the NativePAGE Sample Prep Kit (ThermoFisher Scientific BN2008). PVDF membranes used for blotting were presoaked in 100% ethanol (EtOH), rinsed with MQ H_2_O, then equilibrated for 15 min in 1X transfer buffer with no EtOH (using Trans-Blot Turbo 5x Transfer Buffer, Bio-Rad # 10026938). After transfer using the built-in High Molecular Weight protocol of the Trans-Blot Turbo instrument (1.3 A, 2.5 to 25 V, 10 min), the membranes were fixed in 8% acetic acid. For dye removal, the membranes were air-dried and rinsed with MQ H_2_O until the dye was mostly removed. The Bio-Rad Trans-Blot Turbo Transfer System was used for blotting as outlined above.

#### 2D PAGE and Western blotting

Following NativePAGE performed as above, sample lanes were excised and soaked in 1X SDS loading buffer for 5 min. The gel strips were then placed horizontally at the top of a glass plate sandwich mounted in a standard gel casting apparatus. The plate sandwich contained a polymerized 12% SDS-PAGE separating layer poured to about 5 cm from the top of the plates. After placement of a native gel strip at the top of the plates, a 4% stacking layer was poured to fill the gap between the gel strip and the separating layer and allowed to polymerize. The SDS-PAGE dimension ran at a constant 150 V for 2–2.5 hr. Western blotting was then performed using the Bio-Rad Trans-Blot Turbo Transfer System and PVDF membrane as described above.

#### Proteome analysis

The proteome of six individual stage VI nuclei and six low speed supernatants of cytoplasmic homogenate were determined by liquid chromatography coupled to mass spectrometry (LC-MS) as previously described for whole cells [44]. For the proteins of interest, relative abundance values as % of total ∑#PSMs were ranked using the PERCENTRANK.EXC function in Excel 2016. The source data for Figs 1F and 3A are available in S1 Table.

## Results

### Oocyte isolation and maintenance

Cells at the last two stages of oogenesis were used (stages V and VI [30]). The results did not vary between these cell types. The strategy for oocyte isolation and maintenance is fully described in the Materials and Methods section. Briefly, oocytes were isolated by collagenase digestion of ovary fragments and mechanical removal of investing somatic cells (Fig 1A). The latter step reduces the possibility that the contents of contaminating whole cells affect enzyme behavior in cell fractions [1,45]. Oocytes were maintained in a nutrient-free OR-2 saline solution, in which they remain healthy and competent for induction of genes in transplanted nuclei for four days [46,47]. Oocytes isolated in saline have been used to study whole cell metabolism and to prepare cell extracts for kinetic studies of metabolic enzymes [48,49].

#### GAPDH in the oocyte

Higher eukaryotes express a somatic and spermatogenic form of glyceraldehyde 3-phosphate dehydrogenase (GAPDH and GAPDHS respectively [15]). mRNA-seq data [50] and protein profiles obtained by LC-MS reveal that GAPDH expression in stage V/VI oocytes is limited to the somatic form [31,32]. GAPDH performs its catalytic function as a homotetramer [15]. The predicted 3D structure of a single chain of X. laevis GAPDH generated by RoseTTAFold closely approximates the known structure of monomers in the human homotetramer (Fig 1B). Previous analysis by bottom-up proteomics suggested that like most glycolytic enzymes of oocytes, GAPDH is abundant in the nucleus (S1 Fig, concentration data from reference 31). Indeed, SDS-PAGE and Western blotting revealed robust expression of full-length GAPDH in ensemble samples of whole nuclei and cytoplasms dissected from isolated oocytes (Fig 1C).

In mammalian cells, exogenous signals can trigger translocation of GAPDH from the cytoplasm to the nucleus [51]. Accordingly, we hypothesized that GAPDH is found in the nucleus of isolated oocytes not because that is its physiological location, but because the process of oocyte isolation triggers stress signalling that drives nuclear accumulation. To test this hypothesis, GAPDH abundance was assessed in: 1) nuclei freshly dissected from oocytes in unprocessed ovary fragments (dissection performed under cold oil), and 2) nuclei dissected from oocytes after isolation and overnight culture in saline medium. Western blotting of pooled samples revealed that the nuclear concentration of GAPDH is similar in these cell states (Fig 1D). This finding is consistent with the notion that nuclear localization of GAPDH is a feature of healthy oocytes *in vivo*.

GAPDH monomers exist in different states of self-association, of which only the tetramer is active [52,53]. The different association states of GAPDH can be separated by size exclusion chromatography [54] and native polyacrylamide gel electrophoresis [55]. We analyzed nuclear and cytoplasmic homogenates using native PAGE in conjunction with Western blotting. The goal here was to explore the possibility that oocyte GAPDH exists in different states of self-association (and therefore activity) depending on cell location.

Probing of oocyte homogenates was preceded by a validation experiment in which purified recombinant human GAPDH was resolved in the Invitrogen 4–16% NativePAGE Bis-Tris Gel System. Human GAPDH separated into three distinct bands, which we provisionally identify as monomer, dimer and tetramer (Fig 1E, left panel, lanes 1 and 2). Analysis by two-dimensional native PAGE/SDS PAGE confirmed that the signal in the bands resolved by native PAGE is due to GAPDH, as follows. A strip equivalent to lane 1 in the left panel of Fig 1E was excised from a native gel and placed lengthwise on top of a standard SDS polyacrylamide gel for electrophoresis under denaturing conditions. All the reactivity in the lane from the native gel migrated at approximately 36 kDa by SDS-PAGE (Fig 1E, top image of middle panel). This result is consistent with designation of the immunoreactive bands in the native gels as monomeric and oligomeric forms of human GAPDH.

By native PAGE the GAPDH in oocyte cytoplasmic homogenate comigrated with the provisional human tetramer (Fig 1E, left panel, lane 3). The cytoplasmic GAPDH resolved by native PAGE also ran as a single band by two-dimensional native/SDS PAGE (Fig 1E, bottom image of middle panel). Furthermore, cytoplasmic and nuclear GAPDH of the oocyte comigrated in native gels (Fig 1E, right-most panel). Collectively these findings indicate that the predominant form of GAPDH in the oocyte cytoplasm and nucleus is the tetramer. This assignment of association state is plausible given that the tetramer is the active species of GAPDH [53], and that robust GAPDH activity is detectable in nuclear and cytoplasmic homogenate (section “GAPDH activity assay: whole compartment preparation and activity assay optimization”). Overall, we conclude from this analysis that the cytoplasmic and nuclear pools of GAPDH exist in the same state of oligomerization and that this state corresponds to the tetramer.

In pioneering studies of the oocyte proteome [31,32], and Fig 1C–1E above, oocyte GAPDH has been treated as a single protein species. However, because the evolution of *X*. *laevis* featured whole genome duplication, there are two types of somatic GAPDH in the oocyte. They are encoded by the gapdh.s and gapdh.l genes [50]. The corresponding GAPDH.s and GAPDH.l proteins are closely related (333 amino acids, 91.6% identical and 96.1% similar with no gaps) and both are predicted to be catalytically active. In the oocyte as a whole, GAPDH.s and GAPDH.l are of similar abundance [50,56] and their combined expression is high [31]. Therefore it is plausible that the cytoplasm and nucleus may differ with regard to their content of GAPDH.s and GAPDH.l. We explored this possibility by using LC-MS based proteomics to estimate protein abundance in six individual nuclei and clarified homogenate of six individual cytoplasms. Cytoplasmic homogenates were clarified by centrifugation at 7,500 x g [57]; the resultant supernatant is referred to as S7500. Cross-contamination of nuclear and cytoplasmic S7500 fractions was assessed by analysis of conventional markers of the nucleus and cytoplasm (Fig 1F). Spectral counting was used to estimate protein abundance, with counts scored as % of total Σ# peptide spectral matches (PSMs). Abundance estimates were also ranked for each sample (% rank). The histone chaperone nucleophosmin/nucleoplasmin 2 was abundant in all oocyte nuclei, but not detectable in any S7500 (NPM2 in Fig 1F); note that NPM2 is exclusively nuclear during interphase in human somatic cells and oocytes–see Stelzer et al., 2016 [58]. The cytosolic ATP6V1B2 subunit of the vacuolar H^+^-ATPase [59] was absent from all nuclei but present in all cytoplasmic S7500 preparations. These findings validated the fractionation method and set the stage for analysis of GAPDH.s and GAPDH.l expression as revealed within a sample by the raw value of the Σ# PSMs for each isoform (Fig 1G). Because 96% of total oocyte GAPDH resides in the cytoplasm [43], GAPDH.s and GAPDH.l are expected to be of similar abundance in whole cytoplasm and the whole oocyte. This is the case. The whole oocyte abundance ratio of these proteins from the data in Peshkin et al. (2019) [56] is 0.927. For the cytoplasm, the ratio of GAPDH.s Σ# PSMs/GAPDH.l Σ# PSMs is 1.194 (Fig 1H, right-most bar). More importantly, the GAPDH.s/GAPDH.l abundance ratio is not statistically different between the cytoplasm and nucleus (Fig 1H; 2-tailed Student’s t-Test, 2 sample equal variance, P = 0.7968). Collectively these findings reveal that the cytoplasm and nucleus have the same expression pattern of GAPDH.s and GAPDH.l. It follows that isoform expression pattern will not underlie differences between the cytoplasmic and nuclear pools of GAPDH in activity behavior.

### GAPDH in oocyte metabolism: A working model of compartmentalized activity

Dworkin and Dworkin-Rastl (1989) [49] developed a widely accepted model of oocyte metabolism based on the results of classical tracing experiments in which the cytoplasm and nucleus were treated as one compartment. In this model, ATP synthesis in full grown oocytes is supported mainly by the catabolism of stored yolk proteins. As such, there is little demand for glycolytic synthesis of ATP. This does not mean that flux through glycolytic enzymes is inhibited. Indeed, reverse flux through glycolytic enzymes is essential for glycogen synthesis (it provides carbons for glycogen synthesis).

**Fig 2:**
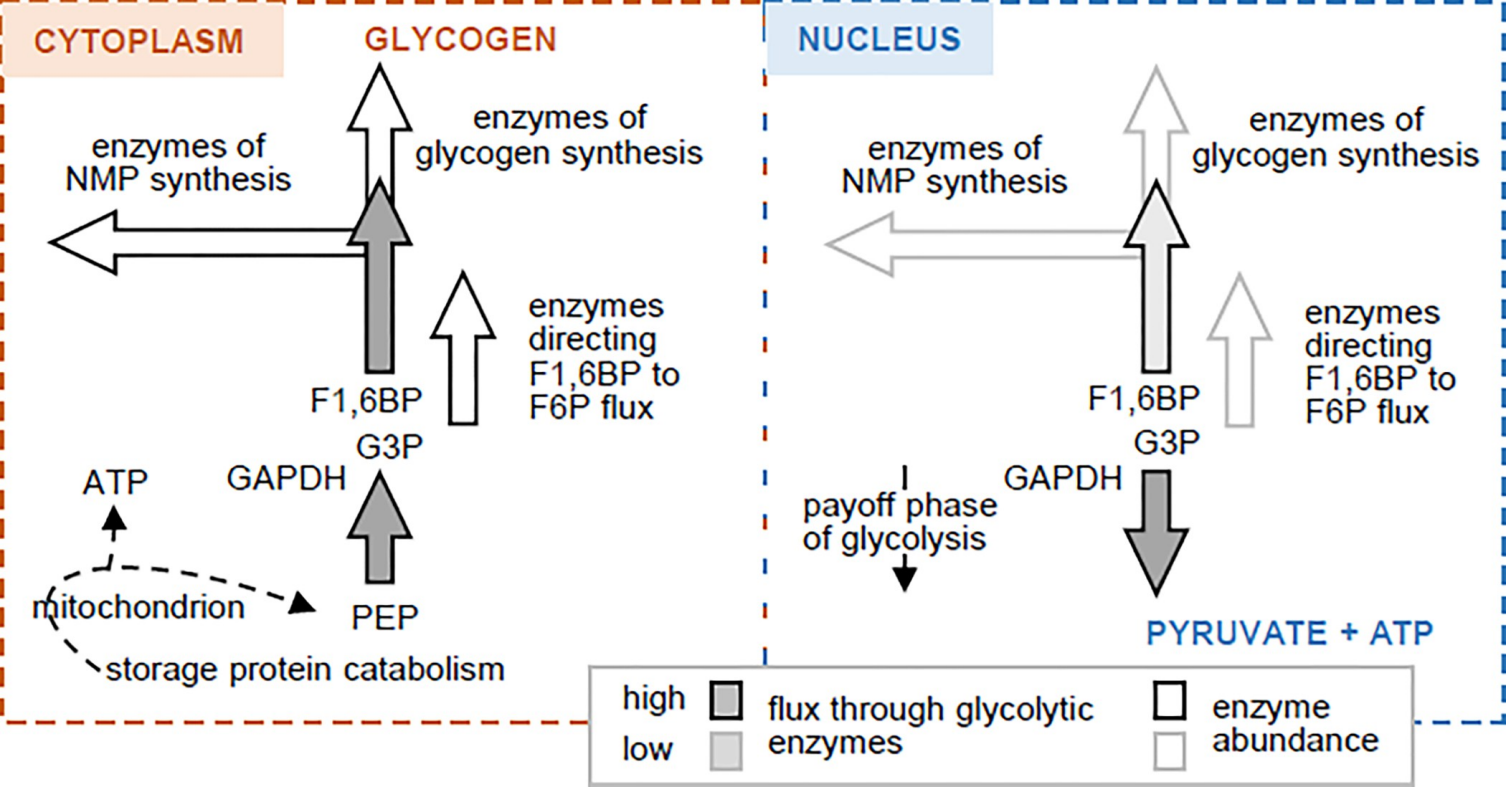
A working model of metabolic flux through glycolytic enzymes located in the nucleus and cytoplasm of full-grown *Xenopus laevis* oocytes. Glycolytic enzymes support opposite directions of flux in the cytoplasm and nucleus. In the cytoplasm, GAPDH activity supports anabolic pathways. The opposite is true in the nucleus, where flux through GAPDH supports ATP synthesis. Grey-filled arrows indicate flux direction and relative intensity (darker shading means higher flux). White-filled arrows represent enzyme abundance; the darker the outline of the arrow, the higher is the enzyme abundance. The white arrows also show flux direction. The dotted line is a multi-enzyme pathway that generates cytosolic PEP and ATP. F1,6BP, fructose 1,6-bisphosphate; G3P, glyceraldehyde 3-phosphate; PEP, phosphoenolpyruvate. See section “GAPDH in oocyte metabolism: A working model of compartmentalized activity” and S1 Fig for details.

Our refinement of the Dworkin and Dworkin-Rastl model proposes that net flux through glycolytic enzymes in whole cell tracer studies is a combination low intensity glycolytic flux in the nucleus that is masked by high reverse flux in the cytoplasm (Fig 2 summarizes the comprehensive model in S1 Fig). This model builds on what is now known about the compartmentalization of enzymes in three pathways: glycolysis, glycogenesis and nucleoside synthesis [31,32,60,61]. It incorporates previous speculations about the operation of nuclear-localized glycolytic enzymes based partly on the finding that phosphofructokinase expression is 2.5-fold lower in the nucleus than the cytosol [31]. The focus of the model is the lower payoff phase of glycolysis, which starts with GAPDH conversion of G3P to BPG.

We propose that the payoff phase of glycolysis is favored in the nucleus because precursors also used for glycogen and nucleotide synthesis are poorly shunted to these pathways in the nucleus (Fig 2; darker fill of arrows corresponds to higher flux). This situation arises because of low expression of key enzymes of glycogen and nucleotide synthesis in the nucleus compared to the cytoplasm (Fig 2; darkness of outline of white-filled arrows reflects level of expression; details in S1 Fig). Glycogen synthase 1 and Carbamoyl-phosphate synthetase 2, Aspartate transcarbamylase, Dihydroorotase (CAD) for example are essentially absent from the nucleus (respectively 0.5 and 3.3 nM; expression ratios between nucleus and cytosol of 1:1880 and 1:173). Overall, we conclude that the nucleus is more permissive than the cytosol for payoff flux through GAPDH and the other enzymes of the lower phase of glycolysis.

**Fig 3:**
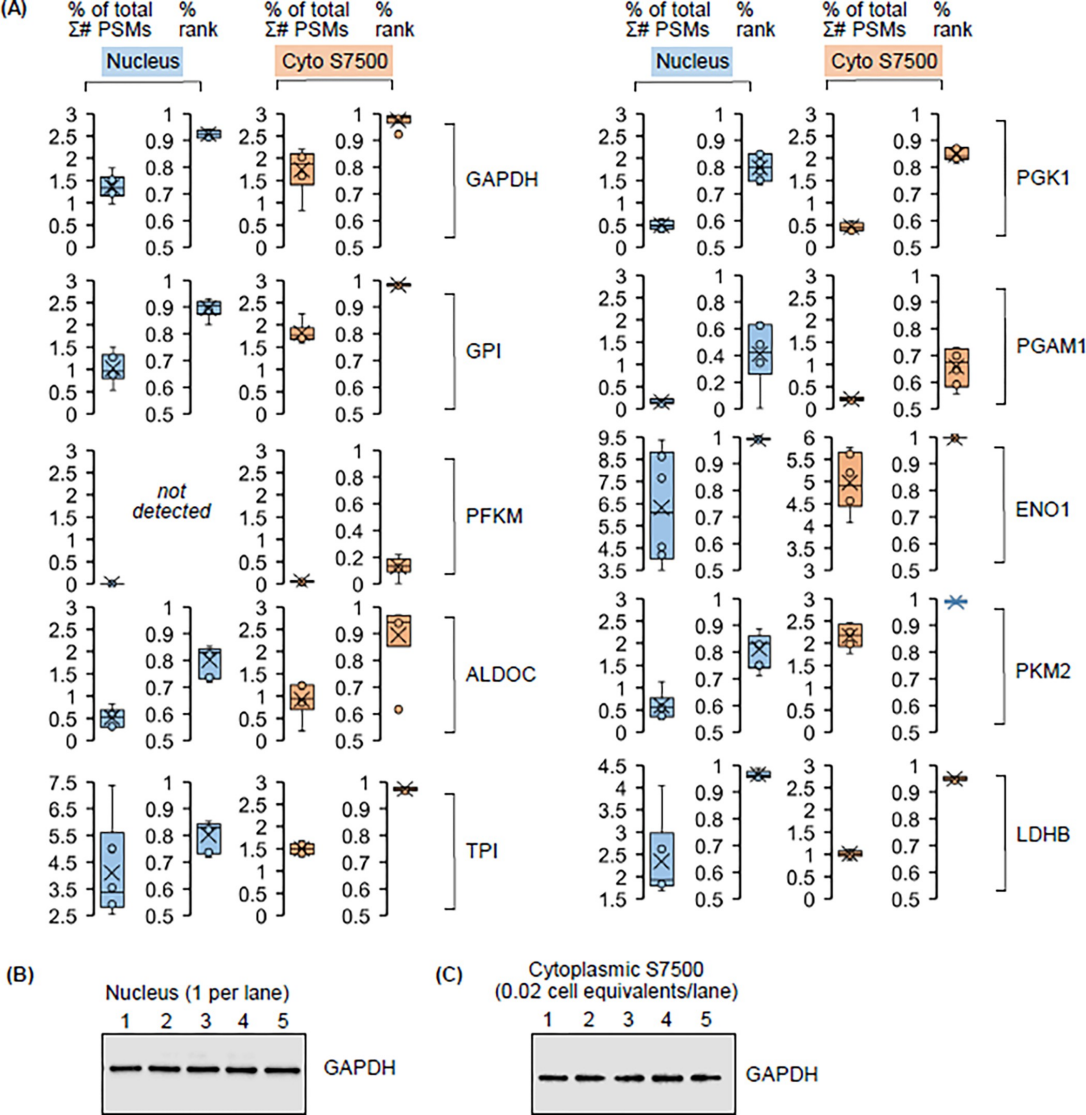
Single cell analysis of enzyme relative abundance in the oocyte nucleus and cytoplasm. (A) Relative abundance of glycolytic enzymes and lactate dehydrogenase in the oocyte nucleus and yolk-free cytoplasm. % of total Σ# PSMs and % rank in each sample as described in Fig 1F. Except for phosphofructokinase PFKM, glycolytic enzymes and LDHB were detected in all nuclei and cytoplasms. (B) Western blotting analysis of GAPDH in five individual nuclei from late stage V cells. (C) Western blotting analysis of GAPDH in S7500s obtained from five individual stage V cytoplasms (same oocyte isolation experiment as panel B).

If the operation of glycolytic enzymes in both the cytoplasm and nucleus is a robust feature of metabolic programming in the oocyte, then their expression profile is expected to be similar between individual nuclei and between individual cytoplasms. To test this prediction, the abundance and abundance rank of glycolytic enzymes was determined (Fig 3A) using the datasets upon which analysis of GAPDH.s and GAPDH.l was based (Fig 1F–1H). Poor expression of hexokinase (HK2, not shown) and phosphofructokinase (PFKM) in the nucleus, as first reported by Kirli et al. [31], was a feature of all individual nuclei (Fig 3A). All other glycolytic enzymes as well as lactate dehydrogenase (LDHB) were detected in all nuclei and cytoplasmic S7500s. GAPDH was readily detected in five nuclei and five cytoplasmic S7500s by Western blotting (Fig 3B and 3C). These findings support the hypothesis that potential nucleus-specific and cytosol-specific programs of metabolism supported by glycolytic enzymes will operate in the majority of oocytes.

### GAPDH activity assay: Validation using purified enzyme

Previous efforts to obtain *in vivo*-like kinetic data for metabolic enzymes [62,63] spurred development of the analytical approach reported here. Three points are noteworthy. First, instead of end-point assays which were typical in early studies, we used kinetic assays of activity. Second, non-physiological product detection reagents were avoided. Rather, enzyme activity was assessed by passive spectrophotometric detection of NADH at 340 nm. Third, the reaction buffer used (HR, homogenization and reaction buffer) reproduces the Mg^2+^, Na^+^, K^+^ and Cl^-^ concentrations of the nucleocytosol and its pH (7.4). It is based on a buffer developed to study structural features of the oocyte nucleus [40,41]. This buffer does not cause actin network gelling which might induce nuclear aggregates containing GAPDH [64].

The suitability of HR buffer for studies of GAPDH kinetics was uncertain because GAPDH activity in crude cell extracts is often assayed at pH 8.5 [15,21]. Furthermore, the pH of HR buffer does not fall within the optimum range reported for the purified enzyme (pH 8.0–8.5 for G3P oxidation) [65]. Therefore, before embarking on a comparison of GAPDH activity in nuclear and cytoplasmic homogenates, we compared the activity of purified GAPDH in HR buffer and a pH 8.5 buffer recommended by a commercial supplier of GAPDH (available at http://www.worthington-biochem.com/GAPD/assay.html) [54,66]. The reaction mix included arsenate to restrict activity to G3P consumption [67] and to inhibit triose phosphate isomerase [68] and pyrophosphatase [69].

**Fig 4:**
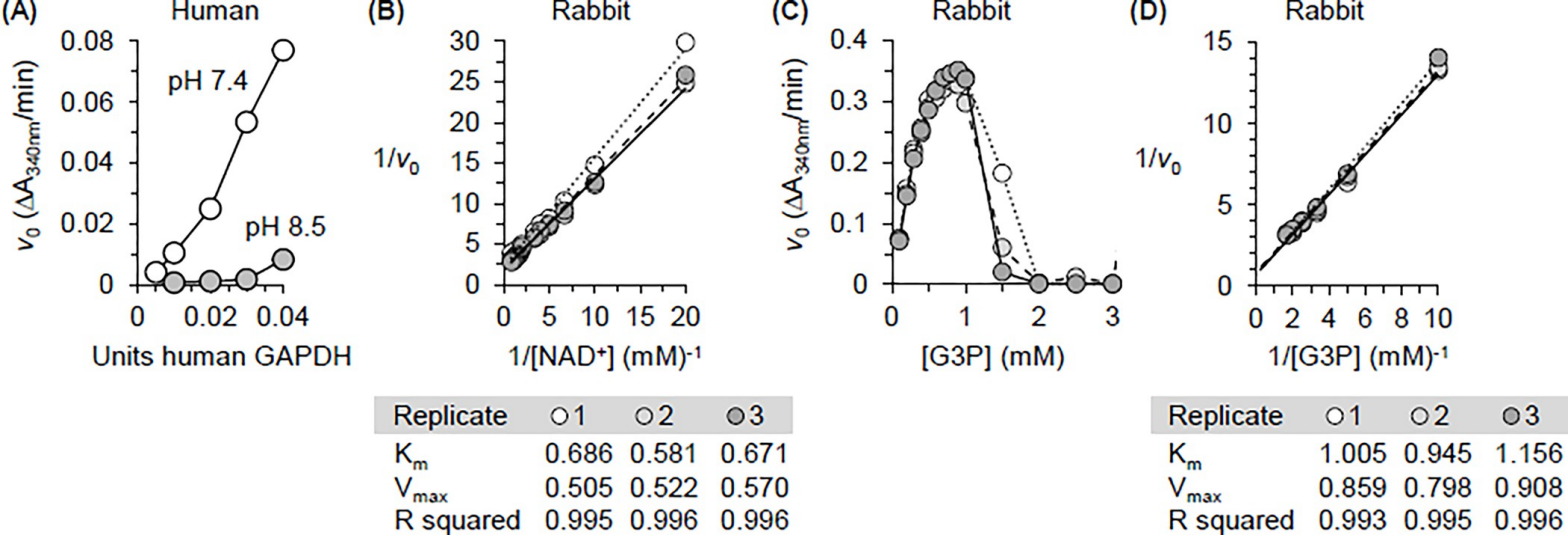
Near-native HR buffer is suitable for the assay of GAPDH activity. (A) Initial rate of recombinant human GAPDH in a standard pH 8.5 buffer (grey-filled circles) and HR buffer, which is adjusted to pH 7.4 (open circles). HR buffer supports high activity of this enzyme preparation. (B) Double reciprocal plot of *v*_0_ versus NAD^+^ concentration for rabbit muscle GAPDH in near-native HR buffer. Positive values for kinetic parameters are obtained when purified rabbit GAPDH is assayed in HR buffer. (C) Plot showing the dependence of *v*_0_ on G3Pconcentration in reactions catalyzed by rabbit GAPDH. Substrate inhibition by G3P, a long-known property of GAPDH, is readily apparent when purified rabbit GAPDH is assayed in HR buffer. (D) Double reciprocal plot of *v*_0_ versus G3P concentration for rabbit muscle GAPDH in near-native HR buffer. Positive values for kinetic parameters are obtained in this buffer. In (B)-(D), the results of three independent assays are shown (each data point is the average of a technical triplicate). The linear trendlines were obtained using Excel. In (B) and (D) the units of K_m_ and V_max_ are mM and ΔA_340_/min respectively.

At all enzyme concentrations tested, the initial rate of recombinant human GAPDH supported by saturating amounts of substrate is higher in HR than in pH 8.5 buffer (Fig 4A). *v*_0_ data for rabbit muscle GAPDH were obtained by titration of NAD^+^ and G3P into reactions based on HR buffer.Lineweaver-Burke plots of the relationship between 1/substrate concentration (S) and 1/*v*_0_ were then used to estimate K_m_ and V_max_. Linear fitting of these plots yielded positive values of K_m_ and V_max_ for NAD^+^ (Fig 4B). Robust substrate inhibition by G3P, a well-documented property of GAPDH [70], was readily apparent in plots of the dependence of *v*_0_ on S (Fig 4C). Lineweaver-Burke plots of the data for G3P concentrations that fall below the onset of substrate inhibition yielded positive values of K_m_ and V_max_ for this substrate (Fig 4D). HR buffer was therefore judged suitable for studies of GAPDH activity in reactions containing whole cell compartments.

#### GAPDH activity assay: Whole compartment preparation and activity assay optimization

Oil-isolated whole nuclei and cytoplasms were homogenized in HR buffer at approximately the same ratio of soluble protein to volume of buffer (from protein measurements in [42]). Physical disruption of compartments was achieved by mild trituration and 5 seconds of vortexing. By visual inspection this method limits homogenization to the amount required for even dispersion of cell material.

**Fig 5:**
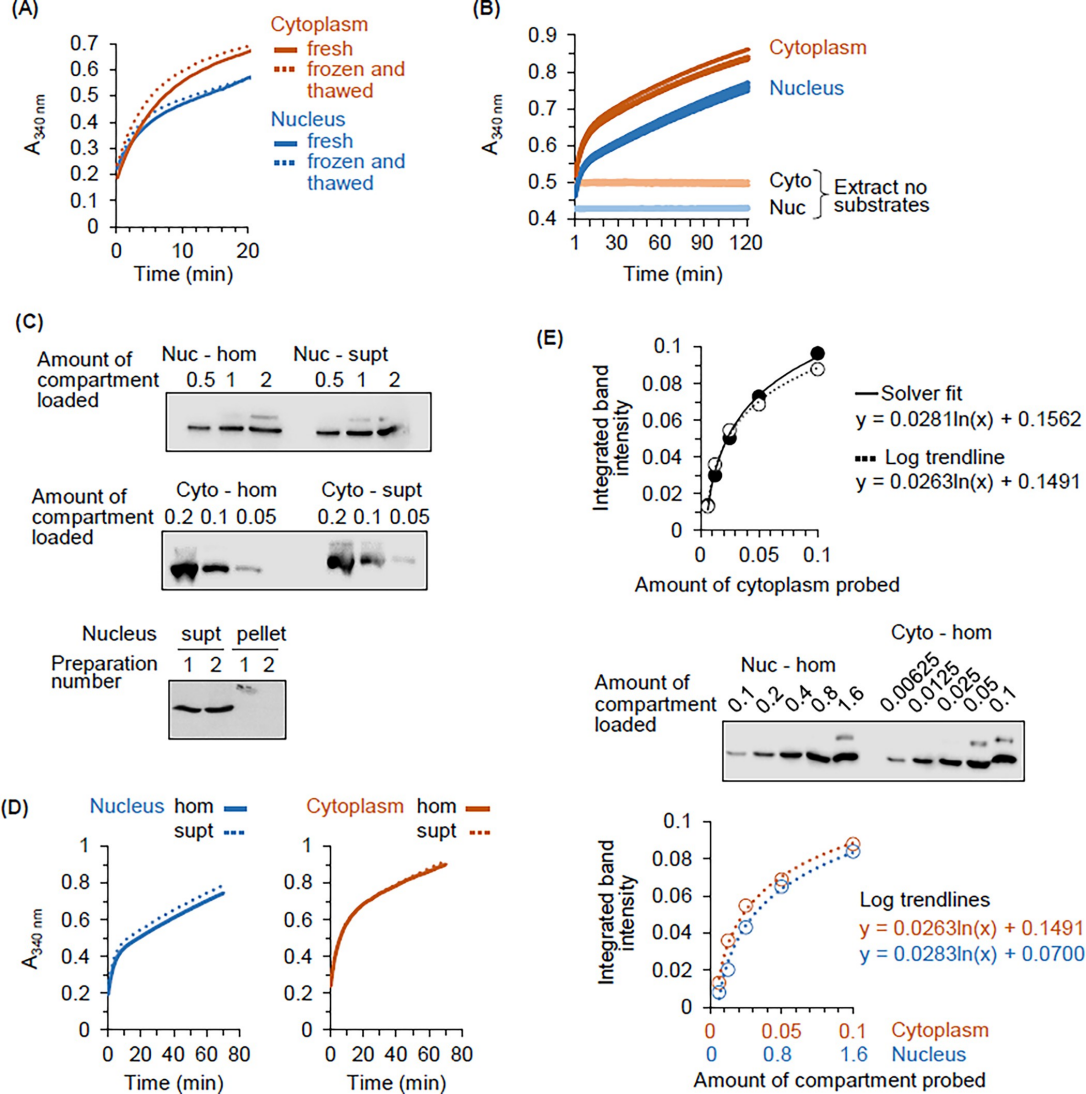
The oocyte system for analysis of GAPDH compartmentalization: Validation of enzyme activity assay and estimation of protein expression by Western blotting. (A) Freeze-thawing does not differentially affect GAPDH activity in whole compartment homogenates. Product accumulation due to GAPDH activity in fresh and frozen-thawed homogenates (solid and dotted lines respectively) of nuclei and cytoplasms (blue and orange respectively). (B) High sampling reproducibility of the GAPDH activity assay. GAPDH activity as product accumulation in replicate samples (n = 6) of a single nuclear and a single cytoplasmic homogenate (blue and orange respectively). The flat line traces are for reactions without substrates. (C) GAPDH is predominantly in the soluble phase of the cytoplasm and nucleus. Top two panels. Western blotting analysis of GAPDH protein in whole homogenates (hom) and matched supernatants (supt) depleted of macromolecular cell features by centrifugation at 15,000 x g. Nuc, nucleus; Cyto, cytoplasm. Bottom panel. Western blotting analysis of GAPDH protein in the supernatant and pellet obtained by centrifugation of nuclear homogenate at 15,000 x g. The experiment was performed in duplicate. (D) GAPDH enzyme activity in whole compartment homogenates is not reduced by removal of macromolecular cell features. GAPDH progress curves for reactions containing nuclear and cytoplasmic fractions as labeled. The fractions are whole homogenate (hom, solid line) and homogenate clarified by centrifugation at 15,000 x g (supt, dashed line). (E) Relative expression of GAPDH in whole homogenates of oocyte nuclei and cytoplasms. GAPDH was detected by chemiluminescent Western blotting. Images captured using the LI-COR system were visualized in Image Studio Lite. The signal intensity data were fitted to hyperbolic curves using the logarithmic trendline and Solver functions in Excel. The top panel compares these fits for a sample of cytoplasmic homogenate. The middle panel is the image of a side-by-side analysis of GAPDH in nuclear and cytoplasmic homogenate. Quantitation of the signal intensity data for this blot is shown in the bottom graph.

A standard protocol for measuring GAPDH oxidative activity in nuclear and cytoplasmic homogenates was developed. In preliminary validation experiments, the homogenate dilutions necessary to obtain linear initial rates were determined under conditions that supported product accumulation for at least one hour only in the presence of both substrates. The standard workflow included lysate thawing after flash freezing and storage at -70°C. A potential drawback of this approach is that freeze/thawing could differentially affect enzyme behaviour in nuclear and cytoplasmic homogenates. To test this hypothesis, fresh nuclear and cytoplasmic homogenates were each split into two aliquots. One was stored on ice while the other was frozen in liquid nitrogen and then thawed. The fresh and frozen/thawed aliquots were then assayed in parallel for GAPDH activity. The effect of freeze-thawing on reaction progress in cytoplasmic and nuclear homogenates was similar in nature. That is, in both homogenate types, freeze-thawing was associated with a slightly elevated rate of product accumulation during the early phase of the reaction (Fig 5A). We conclude that if freeze-thawing does differentially affect the GAPDH activity detected in whole nuclear and whole cytoplasmic homogenates, the effect is modest.

High sampling variability due to the presence of macroscale cell features could confound GAPDH analysis in compartment homogenates. These features include yolk granules, cytoskeletal filaments, nucleoli and chromatin fibers. To test this possibility, GAPDH activity was measured using six aliquots of one nuclear and six aliquots of one cytoplasmic lysate (Fig 5B). The progress curves for each compartment have almost identical shapes and are smooth throughout. The spread between curves in a set is similar, even though nuclear and cytoplasmic homogenates differ greatly in turbidity (Fig 1A, right panel). Therefore, variability between replicate samples of a single primary aliquot is not expected to have a substantial confounding effect when using whole compartment homogenates to measure GAPDH activity by the approach reported here.

Related to the fact that a gentle dispersion method is used, we also tested if GAPDH is predominantly in the soluble or particulate phase of nuclear and cytoplasmic homogenates. Homogenates were centrifuged at 15,000 x g for 15 min. This procedure pellets chromosomes, nucleoli, yolk granules and melanosomes, as well as cytoplasmic assemblages of glycolytic enzymes that form in yeast cells under hypoxic stress [57,71–73]. It did not deplete nuclear or cytoplasmic homogenate of GAPDH protein (Fig 5C, top two panels; compare “hom” to “supt” lanes) and GAPDH abundance was very low in the nuclear pellet compared to the supernatant (Fig 5C, bottom panel). GAPDH activity in nuclear and cytoplasmic homogenates was not depleted by this manipulation (Fig 5D). We conclude that active GAPDH resides mostly in the soluble fraction of the oocyte cytoplasm and nucleus.

We used the same amount of enzyme when determining the ensemble activity behavior of the nuclear and cytoplasmic pools of GAPDH. This was possible because the relative GAPDH content of isolated nuclei and cytoplasms could be estimated by two approaches: 1) using known compartment volumes and concentration data obtained by LC-MS [31] (S1 Fig), and 2) quantitative Western blotting (Fig 5E). The analysis by quantitative Western blotting was not straightforward. The complicating factor was that no combination of protein transfer and imaging conditions yielded data consistent with a linear and proportional relationship between compartment sample amount and GAPDH signal intensity (see also Pillai-Kastoori et al. [74]). However, the data obtained by a variety of blotting procedures did obey nonlinear hyperbolic functions [75–77]. Specifically, robust fitting of the GAPDH data to nonlinear functions was obtained using the natural log trendline and Solver tools in Excel (Fig 5E—top panel compares these fits for cytoplasmic enzyme). Appropriately diluted ensemble samples of nuclear and cytoplasmic homogenate from the same stage VI oocytes, when analyzed on the same blot, yielded very similar natural log trendlines (Fig 5E, middle and bottom panels). From these equations we calculated that 0.044 of one cytoplasm contains same amount of GAPDH as one nucleus. In the present study we adopted a conversion factor (0.04) that is intermediate between the value obtained using quantitative LC-MS (0.038, from data in Kirli et al. [31]) and quantitative Western blotting (0.044, from Fig 5E).

### Comparison of the catalytic behavior of GAPDH in whole nuclear and whole cytoplasmic homogenates

#### Null hypothesis and overview of experimental approach

In this study we test the hypothesis that the nuclear and cytoplasmic pools of GAPDH do not have the same catalytic behavior in whole compartment homogenates under near-native assay conditions. This hypothesis was tested by an approach previously used to develop working models of metabolic wiring from kinetic data obtained by enzyme analysis in cell or tissue homogenates. The work of Arch and Newsholme (1978) [78] exemplifies the approach. These investigators characterized enzymes of adenosine metabolism in whole homogenates of four organs of mouse and rat. For all enzymes in all organs, double reciprocal plots of the dependence of *v*_0_ on substrate concentration yielded positive values for K_m_ and V_max_. Some enzymes differed between organs in their kinetic properties. In rat, for example, the K_m_ of 5’-nucleotidase for AMP was three-fold higher in brain than skeletal muscle. This biochemical information was integrated into a general model of metabolic interaction between organs as it relates to whole animal adenosine metabolism. By extension, we anticipated that comparison of the catalytic behavior of GAPDH in the cytoplasm and nucleus could serve as a starting point for future work aimed at exploring the nature of compartment-specific regulation of the function of metabolic enzymes and its impact (if any) on whole cell metabolism. Here it is appropriate to note a limitation of our approach. To obtain linear initial rates, it was necessary to use highly diluted cell material (see [63]; whole nuclei and cytoplasms at 5000- and 10000-fold dilution respectively). This introduces the possibility that the activity assay is blind to weak intermolecular interactions that may affect the kinetic behavior of GAPDH. To our knowledge, this limitation also applies to all previous kinetic studies of enzymes in crude homogenates.

**Fig 6:**
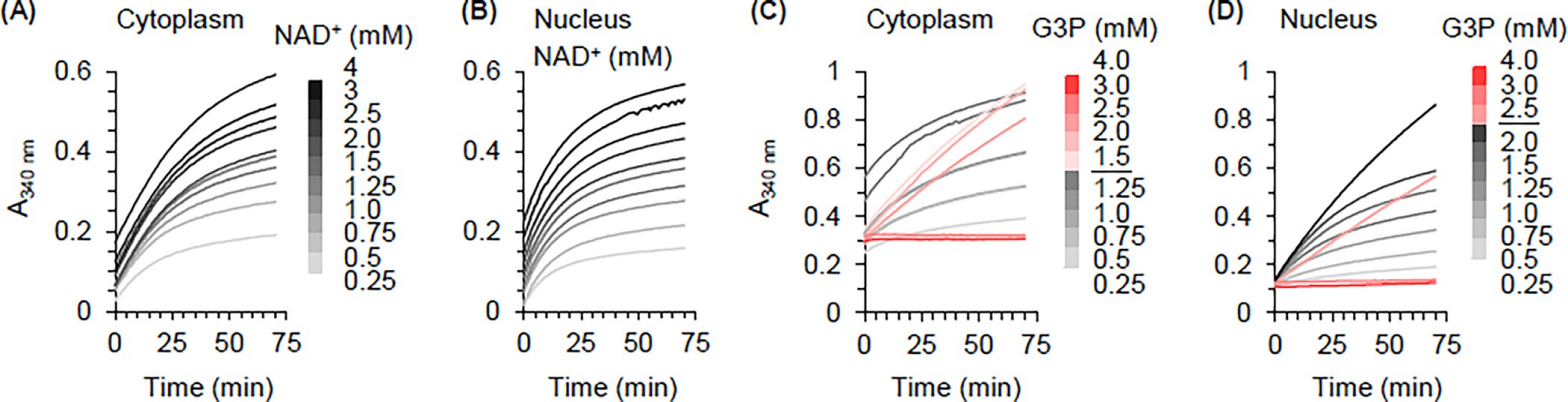
GAPDH activity in representative cytoplasmic and nuclear homogenates. (A) and (B) Progress curves obtained at increasing concentrations of NAD^+^ in cytoplasmic and nuclear homogenate respectively. Identical NAD^+^ concentrations were used for both experiments (key in panel A). (C) and (D) Progress curves obtained at increasing concentrations of G3P in cytoplasmic and nuclear homogenate respectively. The dotted line in the G3P concentration key indicates the titration point beyond which higher G3P concentrations do not cause the reaction rate to increase (red traces). GAPDH activity is fully inhibited at 3 and 4 mM G3P in both nuclear and cytoplasmic homogenate.

Unmatched nuclear and cytoplasmic homogenates were used to develop a standard workflow for assessment of the catalytic properties of nuclear and cytoplasmic GAPDH. Visual assessment of the progress curves for substrate titrations into nuclear and cytoplasmic homogenates suggested that the standard workflow was robust. Approach to saturation for NAD^+^ (Fig 6A and 6B) and inhibition of activity at high concentrations of G3P (red traces in Fig 6C and 6D) were readily apparent for both cytoplasmic and nuclear homogenates.

#### Application of methods for estimating kinetic parameters

In standard enzymology the estimates of kinetic parameters obtained by analyzing homogeneous preparations of an enzyme provide information about the enzyme’s mechanism of catalysis. Such insights are not likely to be gained by assaying GAPDH in whole compartment lysates because these enzyme populations are unlikely to be homogeneous. First, the enzyme in the cytoplasm and nucleus may not be homogeneous with regard to activity state because (for example) some tetramers are acetylated and some are not (20). Second, the proportion of tetramers in different activity states may vary between the compartments. Accordingly, activity signatures obtained by whole compartment enzyme analysis are not interpreted to reflect the mechanistic properties of homogeneous pools of GAPDH [79]. Rather, they are compared to test the hypothesis that the cytoplasm and nucleus are distinct with regard to the whole compartment behaviour of GAPDH as a metabolic enzyme.

The GAPDH activity signature of nuclear and cytoplasmic homogenates was obtained from plots of *v*_0_ dependence on substrate concentration. The signature has two features, namely the shape of the plot of *v*_0_ dependence on substrate concentration, and mathematical fit to Michaelis-Menten behavior. 1. Plot shape was classified by visual inspection as being either hyperbolic or sigmoidal. 2. Fit to Michaelis-Menten behavior. Linear equations were obtained for plots of 1/*v*_0_ versus 1/S, S/*v*_0_ versus S and *v*_0_ versus *v*_0_ /S (Lineweaver-Burke, Hanes-Woolf and Eadie-Hofstee methods respectively) [62]. The equations were assigned to two classes: “valid” or “invalid”. Equations in the valid class yielded positive estimates for kinetic parameters. The corresponding enzyme pools therefore exhibited Michaelis-Menten-like behavior. Equations in the invalid class did not yield positive estimates for kinetic parameters (negative estimates are invalid). This qualitative scoring reflects the behaviour of GAPDH in whole compartment environments. Note that the homogenates for which GAPDH activity deviated from Michaelis-Menten-like behavior were as active overall as homogenates for which positive estimates of K_m_ and V_max_ could be obtained.

#### The activity behavior of nuclear and cytoplasmic GAPDH

**Fig 7:**
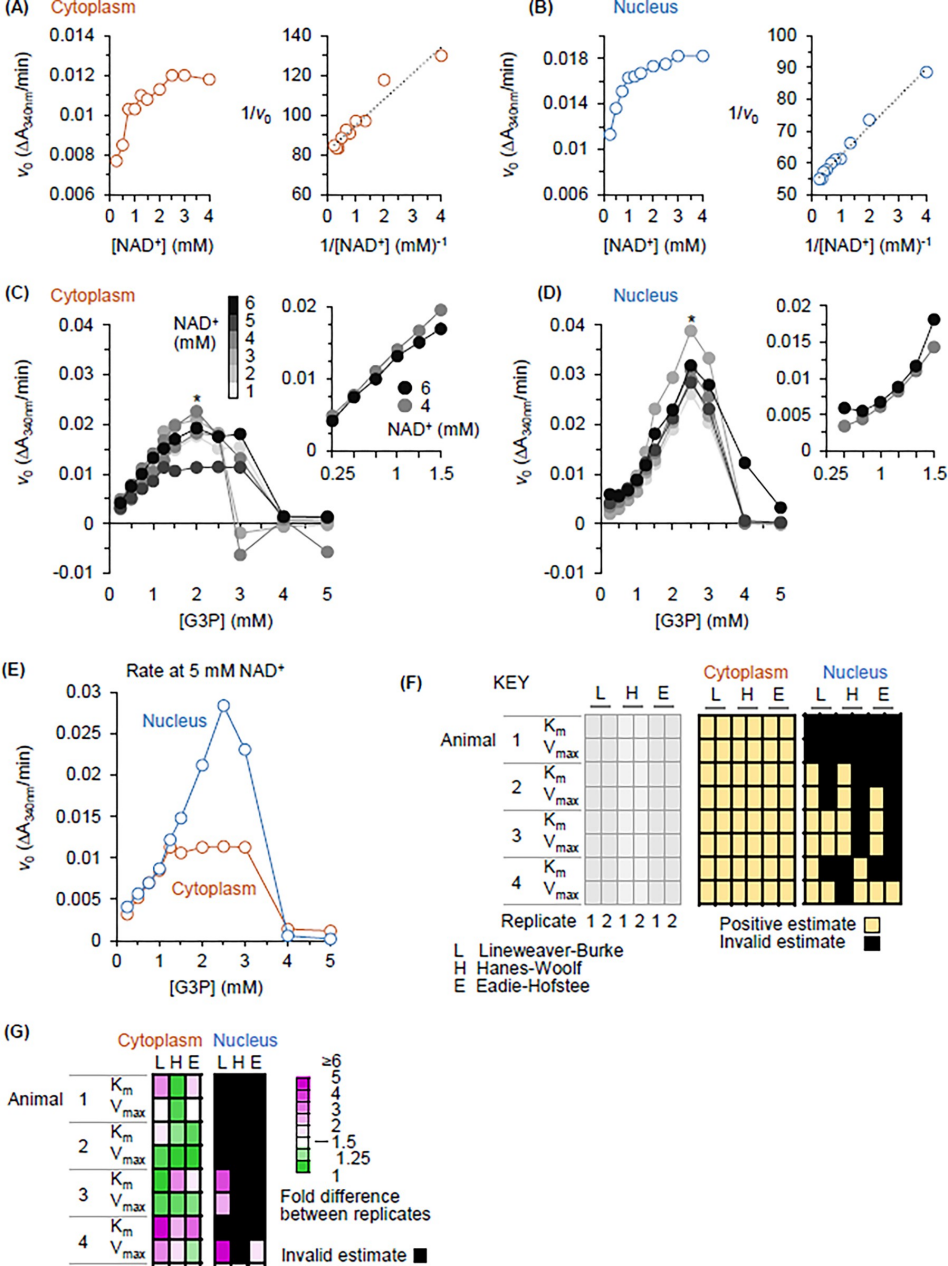
The activity behavior of GAPDH differs between homogenates of whole cytoplasm and whole nucleus. (A) and (B) Michaelis Menten-like behaviour of cytoplasmic and nuclear GAPDH in assays of *v*_0_ versus NAD^+^ concentration. (A) Plot for cytoplasmic homogenate of the dependence of GAPDH *v*_0_ on NAD^+^ concentration (left panel) and Lineweaver-Burke plot of this data (right panel). (B) Plot for nuclear homogenate of the dependence of GAPDH *v*_0_ on NAD^+^ concentration (left panel) and Lineweaver-Burke plot of this data (right panel). (C)—(F) Departure of nuclear GAPDH from Michaelis Menten-like behaviour in assays of *v*_0_ versus G3P concentration. (C) Plots for cytoplasmic homogenate of the dependence of GAPDH *v*_0_ on G3P concentration at 6 concentrations of NAD^+^ (large graph at left). The pullout shows the data at 4 and 6 mM of NAD^+^ over the 0.25–1.5 mM range of G3P concentrations. The latter plots have a hyperbolic (Michaelis-Menten-like) character. (D) Plots for nuclear homogenate of the dependence of GAPDH *v*_0_ on G3P concentration at 6 concentrations of NAD^+^ (large graph at left). The pullout shows the data at 4 and 6 mM of NAD^+^ over the 0.25–1.5 mM range of G3P concentrations. The latter plots have an unexpected sigmoidal character. (E) Direct comparison of the G3P *v*_0_ data for nuclear and cytoplasmic GAPDH at 5 mM NAD^+^. At the peak for both (2.5 mM G3P), *v*_0_ is 2.5-fold higher in nuclear than cytoplasmic homogenate. (F) Color-coded diagram scoring the fit of the *v*_0_ versus [G3P] data to the Michaelis-Menten equation. The fitting methods were Lineweaver-Burke (L), Hanes-Woolf (H) and Eadie-Hofstee (E). Yellow fill indicates that a positive estimate was obtained for the indicated parameter (K_m_ or V_max_). Black fill indicates an invalid estimate. The results are from assays of two independent starting homogenates from four animals. (G) Heatmap highlighting the fold-difference between estimates of kinetic parameters obtained by the Lineweaver-Burke (L), Hanes-Woolf (H) and Eadie-Hofstee (E) methods.

We determined the dependence of *v*_0_ on NAD^+^ and G3P concentration using unmatched and matched cytoplasmic and nuclear homogenates (matched sets being from the same pool of stage VI oocytes of a single animal). Homogenate amounts contained the same quantities of GAPDH protein, as described under “GAPDH activity assays: whole compartment preparation and activity assay optimization”. Representative plots for NAD^+^ are shown in Fig 7A and 7B. By visual inspection the shape of the plots generated using cytoplasmic and nuclear homogenate was similar (left panel of Fig 7A and 7B). The profile was consistent with a relationship described by a rectangular hyperbola. Indeed, a strong linear relationship between 1/[NAD^+^] and 1/*v*_0_ was observed for both cytoplasmic and nuclear GAPDH (right panel of Fig 7A and 7B).

Fig 7C and 7D show data from a complementary experiment in which the dependence of *v*_0_ on G3P concentration was determined at multiple concentrations of NAD^+^. This experiment was performed using matched cytoplasmic and nuclear homogenates. The compartment profiles are similar with respect to substrate inhibition. That is, regardless of NAD^+^ concentration, there is no GAPDH activity at 4 and 5 mM G3P (leftmost plot in Fig 7C and 7D; inhibition by G3P was also observed for unmatched homogenates at a single NAD^+^ concentration–red traces in Fig 6C and 6D).

The cytoplasmic and nuclear profiles diverge, however, over the range of G3P concentrations associated with increasing GAPDH activity. In the plots compiling the data at all NAD^+^ concentrations this difference manifests as convergence on a hyperbolic (Michaelis-Menten-like) profile in the cytoplasm but a sigmoidal profile in the nucleus (graphs at left in Fig 7C and 7D). This difference is clearly apparent at higher concentrations of NAD^+^ over the 0.25–1.5 mM range of G3P concentrations (pullouts in Fig 7C and 7D, *v*_0_ at 4 and 6 mM NAD^+^).

The activity profiles also diverge over the range between 1 and 4 mM G3P. In nuclear homogenate, *v*_0_ clearly peaks at 2.5 mM G3P at all concentrations of NAD^+^ (Fig 7D, left plot). In cytoplasmic homogenate, on the other hand, the profile does not have a sharp *v*_0_ peak (Fig 7C, left plot). This v_0_ difference at 5 mM NAD^+^ (2.5-fold) is highlighted in Fig 7E.

Finally, the maximum *v*_0_ supported in nuclear homogenate is 1.72-fold higher than the maximum recorded for GAPDH in cytoplasmic homogenate (asterisks in Fig 7C and 7D; nucleus = 0.0388 ΔA_340nm_/min at 2.5 mM G3P, 3 mM NAD ^+^ compared to cytoplasm = 0.0226 ΔA_340nm_/min at 2 mM G3P, 4 mM NAD^+^).

These observations are consistent with the hypothesis that the activity behavior of the cytoplasmic and nuclear pools of GAPDH differs when the enzyme is assayed in whole compartment homogenates under near-native assay conditions. In particular, there are no conditions in cytoplasmic homogenate that can support the maximum rate observed for GAPDH in nuclear homogenate. The latter finding rules out an interpretation of the data related to possible assay interference by triose phosphate isomerase (TPI1). TPI1 interconverts G3P and dihydroxyacetone phosphate. The TPI1 dimer/GAPDH tetramer expression ratio is higher in the oocyte nucleus than the cytoplasm (3 and 1.6 respectively; from data in Kirli et al. [31]). If TPI1 somehow depletes G3P in vitro and this is the only difference between the nuclear and cytoplasmic homogenates that affects GAPDH activity, then the cytoplasmic pool would have higher activity than the nuclear pool. However, the reverse is true (Fig 7E).

The results in Fig 7A–7E set the stage for a more comprehensive analysis of matched nuclear and cytoplasmic homogenates from stage VI oocytes. For this analysis we collected data using homogenates prepared from oocytes isolated from four animals. For each animal, cytoplasms and nuclei were processed in succession over a 2-hour period. Oocytes were dissected under oil at room temperature. To prepare nuclear homogenate, oocytes were dissected in groups until 10–15 nuclei had been obtained (this required 5–10 minutes). The 10–15 nuclei were then collected as a group and transferred to chilled oil (6°C). The process was repeated until 72 nuclei had been accumulated. These 72 nuclei were then homogenized in ice cold HR buffer to obtain the starting nuclear homogenate. Cytoplasmic homogenate was prepared by a similar approach, except that starting homogenates were prepared from 40 cytoplasms. Cell dissection was performed at room temperature because it was difficult to obtain intact nuclei when dissection was performed at 6°C. Starting homogenates were frozen in multiple primary aliquots. Each activity analysis was performed twice using different primary aliquots of the same starting homogenate. The data points of progress curves were the averages of three technical replicates generated using subsamples of the same primary aliquot. To summarize: 1) for activity profiling of material from each animal, two replicates were performed (each is for a separate starting homogenate; see Key, Fig 7F), and 2) the data for each replicate was based on triplicate determinations of *v*_0_ at each point in a substrate titration. This comprehensive analysis had an unexpected outcome. Cytoplasmic homogenates yielded reproducible Michaelis-Menten-like profiles of GAPDH activity. In nuclear homogenates on the other hand, GAPDH activity behavior was a) divergent between replicate starting homogenates, and b) not consistent with Michaelis-Menten-like activity. The results supporting these conclusions are presented in a color-coded diagram (Fig 7F) and a heatmap (Fig 7G).

For all assays of cytoplasmic GAPDH, fitting of the plots obtained by the Lineweaver-Burke, Hanes-Woolf and Eadie-Hofstee linearization strategies all generated valid line equations from which positive estimates of apparent K_m_ and V_max_ could be calculated (Fig 7F, Cytoplasm–yellow shading). This result was not obtained for nuclear GAPDH (Fig 7F, Nucleus). The nuclear enzyme diverged from the cytoplasmic enzyme in two ways. First, positive K_m_ and V_max_ estimates could not be obtained for all replicates of all nuclear homogenates. For example, regardless of the linearization method used, both replicates of the nuclear homogenate of Animal 1 scored as yielding invalid K_m_ and V_max_ estimates (black boxes in Fig 7F). Second, the replicates of a primary nuclear homogenate could differ in scoring for fit to Michaelis-Menten behavior. Consider the results for Animal 2 based on the Lineweaver-Burke and Hanes-Woolf plotting strategies (Fig 7F). Replicate 1 scored positive for K_m_ and V_max_, while the scores obtained for replicate 2 were invalid.

The heatmap in Fig 7G more fully illustrates divergence of the estimates of K_m_ and V_max_ between replicates. The value represented in each heatmap cell is the larger estimate for a parameter divided by the lower estimate (fold difference between replicates). Because all replicates of cytoplasmic GAPDH yielded positive estimates for K_m_ and V_max_, there were no invalid estimates of the fold difference between replicates. And for three of the four animals, a difference ratio close to 1 was obtained using one of the linearization strategies (Animal 1 and 2 –Hanes-Woolf; Animal 3 –Lineweaver-Burke). The overall pattern of divergence between replicates was very different for nuclear homogenates. First, a meaningful estimate of fold difference between replicates could not be obtained for the nuclear homogenates of Animals 1 and 2 (Fig 7G, black-filled boxes). And while a meaningful fold-difference between replicates was obtained for K_m_ and V_max_ of Animal 3 (Lineweaver-Burke linearization), the difference was higher than obtained for replicates of cytoplasmic homogenates regardless of the linearization method used to estimate kinetic parameters (numerical data in S2 Table). To summarize, comparative analysis of enzyme behavior has captured an unexpected axis of biochemical heterogeneity that applies to GAPDH in samples of nuclear but not cytoplasmic homogenate. Overall, this finding is consistent with the hypothesis that compartmentalization of GAPDH protein between the cytoplasm and nucleus is associated with divergence of its activity regulation *in vivo*.

## Discussion

Here we built on conventional methods for characterizing purified enzymes [63,80] to assess the activity behavior of GAPDH in whole compartment homogenates of the cytoplasm and nucleus. Enzyme assays were normalized for enzyme protein amount and performed in an *in vivo*-like homogenization and reaction buffer. GAPDH in reactions containing all the constituents of native nucleus did not have the same behavior signature as in reactions containing all the constituents of native cytoplasm. The strongest divergence was revealed in assays of the dependence of *v*_0_ on the concentration of the substrate G3P (Fig 7C–7G, S2 Table).

In all cytoplasmic homogenates tested, the equation of a rectangular hyperbola described the relationship between the *v*_0_ of GAPDH and [G3P]. Positive estimates for apparent K_m_ and apparent V_max_ were obtained, and their values did not substantially fluctuate between replicate experiments. For a purified enzyme, this would be classical Michaelis-Menten behavior.

This outcome was not obtained for GAPDH in whole nuclear homogenate. In nuclear homogenate, the dependence of *v*_0_ on G3P concentration was not described by a rectangular hyperbola (the plots had a sigmoidal profile) and it was the exception to obtain positive values for apparent K_m_ and apparent V_max_. When estimates of K_m_ and V_max_ could be obtained for nuclear GAPDH, they differed substantially from the estimates obtained for GAPDH in matched cytoplasmic homogenate (S2 Table). Therefore, nuclear and cytoplasmic homogenates are distinctive in terms of their support of GAPDH catalytic activity. This divergence could reflect a difference in the enzyme’s modification state or interaction with allosteric effectors (see references in Introduction).

The variability of GAPDH behavior between separately prepared starting homogenates of nuclei was unexpected and raises an important question: was a step in homogenate preparation potentially permissive for variable amounts of biochemical drift between starting homogenates? The answer is yes. Each starting nuclear homogenate was from a set of 72 cells. Because recovery of intact nuclei from chilled cells has a high failure rate, subsets of cells were dissected and pooled at room temperature before compartment transfer to oil at 6°C (see Materials and Methods; note that preparation of cytoplasmic homogenate also involved room temperature processing). The duration of the room temperature step varied between 5 and 10 min depending on how easily each cell in a set was dissected. Variability of GAPDH behavior between starting nuclear homogenates (sets of 72 nuclei) was likely introduced during this time. Processing of a metabolic effector is one possible source of this *in vitro* variability [81]. In future studies a starting point for exploration of this idea would be to include analysis of homogenates prepared at 6°C from start to finish. Finally, we note that the “weak point” of our approach has exposed a potential mechanism of GAPDH regulSation that could be fully revealed by work involving compartment metabolomics and kinetic analysis of purified enzyme in the presence and absence of candidate allosteric regulators. As in the present report, the broad working hypothesis for these studies would be that the distinctive behavior of GAPDH in nuclear and cytoplasmic homogenates reflects the existence of a cellular mechanism for differential control of the activity of the nuclear and cytoplasmic pools of GAPDH.

In what ways might the nuclear and cytoplasmic pools of GAPDH contribute differently to oocyte physiology and be differentially regulated to integrate these activities into the whole cell program of metabolism? One possibility is that the global abundance of some key intermediates of central metabolism is determined predominantly by the biochemical programming of nuclear rather than cytoplasmic GAPDH. We consider this unlikely because in the full-grown oocyte only 4% of all GAPDH is found in the nucleus [43]. It follows that nucleus-specific tuning of GAPDH activity would not directly dictate the running of modules of metabolism located only in the cytoplasm (glycogen and NMP synthesis, Fig 2). Rather, the purpose of nucleus-specific modulation of GAPDH activity might be to ensure an appropriate supply of metabolites for use by proteins that control non-metabolic nuclear activities. One metabolite in this GAPDH-dependent supply model is ATP. Its synthesis by pyruvate kinase in the nucleus is expected to require nuclear GAPDH activity [31]. ATP produced by way of nuclear GAPDH and PKM2 might help to sustain chromatin remodeling by DNA-dependent ATPases. This remodeling is important for DNA transcription, replication, recombination and repair [82–85]. ATP produced in the nucleus might also partly support helicase activity that is essential for pre-mRNA splicing [86]. Since NAD^+^ and NADH are interconverted by GAPDH, nucleus-specific regulation of GAPDH could also buffer the NAD^+^/NADH ratio in the nucleus against fluctuations that occur in the cytoplasm. This buffering might ensure appropriate operation of NAD^+^-dependent lysine deacetylases that function in concert with lysine acetylases to modulate the turnover rate of histone acetylation in chromatin [87].

Our study focused on female germline cells. Considering that studies of GAPDH in various mammalian somatic cell types have revealed multiple mechanisms by which its activity can be regulated (see Introduction), a divergence of GAPDH activity regulation between the nucleus and cytoplasm of somatic cells would not be surprising. In normal human, keratinocytes in the stratum spinosum and basale of the epidermis and glandular cells of the stomach epithelium are examples of cell types for which immunohistochemical data are consistent with very high GAPDH expression in the nucleus [88]. In these cells it is possible that overall flux through GAPDH is modulated by the catalytic behavior of the nuclear pool of enzymes.

More broadly, what has been learned about GAPDH leads us to speculate that other enzymes of central metabolism with a pool in the nucleus and the cytoplasm might also have different activity profiles in these compartments. Of interest in the oocyte are malate dehydrogenase 1, isocitrate dehydrogenase 1, glucose 6-phosphate dehydrogenase, 6-phosphogluconate dehydrogenase, enolase, pyruvate kinase and lactate dehydrogenase (respectively MDH1, IDH1, G6PD, PGD, ENO1, PKM2 and LDHA and LDHB). All have a concentration greater than 180 nM in both the nucleus and cytoplasm (S3 Table). Their expression at full length in the nucleus was confirmed by Western blotting (except for LDHA/B, owing to lack of a suitable antibody; S3–S10 Figs). And their activity was readily detected in nuclear and cytoplasmic homogenates (S3–S10 Figs). For example, high activity was detected for PKM2 (S9 Fig) which has the lowest relative concentration in the nucleus of all enzymes tested (1894.4 nM versus 8902.7 nM in cytoplasm, ratio of 0.214). These findings provide a robust starting point for a comprehensive analysis of the activity behavior of the nuclear and cytoplasmic pools of metabolic enzymes in the oocyte. Like GAPDH, many of these enzymes have been reported to occur in the nucleus of mammalian cells [11]. Whole compartment activity analysis as described here for the oocyte may inform studies aimed at assessing the significance of locating catalytically active metabolic enzymes in the nucleoplasm of mammalian cells.

## Supporting information

S1 Fig A working model of metabolic flux through enzymes of glycolysis located in the nucleus and cytoplasm of oocytes. Flux through glycolytic enzymes (circled steps 2–11) occurs in opposite directions in the cytoplasm and nucleus (orange and blue arrows respectively). GAPDH acts at step 6. Relative flux intensity is indicated by line width. Dotted lines are pathways for which some steps have been omitted. Lines with black arrows identify reactions that are expected to operate similarly in the cytoplasm and nucleus. The orange and blue numbers are enzyme concentrations in nM (Kirli et al., 2015). In the cytoplasm, glycogen and nucleoside synthesis (orange boxes, dark outline) predominate over pyruvate synthesis. In the nucleus, flux through GAPDH supports operation of the payoff phase of glycolysis culminating in ATP and pyruvate synthesis (blue box, dark outline). Flux in the opposite direction is limited by low nuclear expression of critical enzymes of glycogen and nucleoside synthesis. The enzymes of glycogen synthesis shown are glycogen synthase 1 (GSY1) and UDP-glucose pyrophosphorylase (UGP2). The enzymes of nucleoside monophosphate (NMP) synthesis shown are: Carbamoyl-phosphate synthetase 2, Aspartate transcarbamylase, Dihydroorotase (CAD); Phosphoribosyl pyrophosphate synthetases 1 and 2 (PRPS1, PRPS2); Uridine monophosphate synthetase (UMPS); Phosphoribosylglycinamide formyltransferase and synthetase, Phosphoribosyl-aminoimidazole synthetase (GART). Glucose input to G6P is not shown. Most ATP production in the oocyte is fueled by mitochondrial metabolism of amino acids derived from vitellogenin. Kırlı K, Karaca S, Dehne HJ, et al. A deep proteomics perspective on CRM1-mediated nuclear export and nucleocytoplasmic partitioning. *eLife*. 2015;4:e11466.
(PDF)

S2 Fig Estimation of amount of cytoplasm associated with oil isolated nuclei. A challenge in biochemical studies of nuclear enzymes is that isolated nuclei can be associated with cytoplasmic material. This material can be in the form of cytoplasm adhering to the nuclear exterior and cytoplasm present in invaginations of the nuclear envelope (which form a “nucleoplasmic reticulum”; Drozdz and Vaux, 2017). To our knowledge the amount of such cytoplasmic material associated with nuclei isolated from tissue culture cells has not been estimated. Such an estimate can however be made for the oil-isolated *X*. *laevis* oocyte (in the diagram the nucleoplasmic reticulum has been enlarged for clarity). This estimate is based on the known volumes of the nucleus and cytosol of stage VI oocytes (Gurdon and Wickens, 1983), and images in two studies of the *X*. *laevis* oocyte. The first reveals the nucleoplasmic reticulum in an equatorial section of a stage VI oocyte (plate 1 in Hausen and Riebesell, 1991; link available at https://www.xenbase.org/entry/doNewsRead.do?id=613). The section was stained with a classical dye that colors the cytosol blue. Penetrations of cytoplasm into the nucleus are evident. Using a high-resolution download of this image, we employed the “point hit” method (Elias and Hyde, 1983) to estimate the volume of the nuclear interior that is cytoplasm. That volume is 1.22 nL. The second image is a transmission electron micrograph showing the rim of cytoplasmic material of an oil-isolated nucleus (Paine et al., 1992). From this image we can estimate the maximum depth of the cytoplasmic rim (0.5 mm) and the corresponding volume of the cytoplasmic rim (0.28 nL). The volume of cytoplasm contributed to oil isolated nuclei by the nucleoplasmic reticulum and cytoplasmic rim is therefore approximately 1.5 nL. Based on this estimate and the concentration of GAPDH reported for oocyte cytoplasm (Kirli et al. (2015), the nuclear concentration of GAPDH would be 4.8135 nM if the only source of the GAPDH was nucleus-associated cytoplasm. The actual nuclear concentration of GAPDH (4851 nM) is however 1000-fold higher than this estimate. Therefore, only approximately 0.01% of GAPDH associated with the oil-isolated nucleus will be contributed as associated cytoplasm. Drozdz MM, Vaux DJ. Shared mechanisms in physiological and pathological nucleoplasmic reticulum formation. *Nucleus*. 2017;8:34–45. Gurdon JB, Wickens MP. The use of Xenopus oocytes for the expression of cloned genes. *Methods Enzymol*. 1983;101:370–386. Hausen P, Riebesell M. 1991. *The Early Development of Xenopus laevis*: *An Atlas of the Histology*. Springer Verlag;1991. Elias H, Hyde DM. *A Guide to Practical Stereology*. Karger Publishers;1983. Paine PL, Johnson ME, Lau YT, Tluczek LJ, Miller DS. The oocyte nucleus isolated in oil retains in vivo structure and functions. *Biotechniques*. 1992;13:238–246. Kırlı K, Karaca S, Dehne HJ, et al. A deep proteomics perspective on CRM1-mediated nuclear export and nucleocytoplasmic partitioning. *eLife*. 2015;4:e11466.
(PDF)

S3 Fig Malate dehydrogenase 1 (MDH1) in nuclear and cytoplasmic homogenates of *X*. *laevis* oocytes. Human UniProt link in Kirli et al., 2015: P40925 · MDHC_HUMAN. Malate dehydrogenase activity in homogenates of whole cytoplasms and whole nuclei (whole cell activity was previously characterized by Gill and Schultz (2022), who also reported on the expression of full-length MDH1 in these compartments). In the present study enzyme activity was analyzed as described for whole oocyte homogenates by Gill and Schultz (2022). Gill GS, Schultz MC. Multienzyme activity profiling for evaluation of cell-to-cell variability of metabolic state. *FASEB BioAdv*. 2022;4:709–723.
(PDF)

S4 Fig Isocitrate dehydrogenase 1 (IDH1) in nuclear and cytoplasmic homogenates of *X*. *laevis* oocytes. Human UniProt link in Kirli et al., 2015: O75874 · IDHC_HUMAN. (A) Expression. Full-length IDH1 protein is present in the oocyte nucleus and cytoplasm. Proteins were resolved by SDS-PAGE and IDH1 was detected by Western blotting using rabbit polyclonal antibody # ARP54787_P050 (Aviva Systems Biology). Migration of the 50 kDa molecular weight marker is indicated at the left. Antibody dilutions: primary 1:2000 and secondary 1:4000. (B) Activity. Isocitrate dehydrogenase activity is detectable in homogenate of whole cytoplasms and nuclei. Assay performed according to standard method for GAPDH. Final substrate concentrations: 2 mM isocitric acid, 1 mM NADP^+^. The product detected is NADPH. (C) Activity: dependence on NADP^+^ concentration. Dependence of the velocity of the isocitrate dehydrogenase reaction in nuclear homogenate on the concentration of added NADP^+^. Each reaction contained the amount of homogenate equivalent to one nucleus. *v* was estimated from the 5–18 min data points of progress curves.
(PDF)

S5 Fig Glucose-6-phosphate dehydrogenase (G6PD) in nuclear and cytoplasmic homogenates of *X*. *laevis* oocytes. Human UniProt link in Kirli et al., 2015: P11413 · G6PD_HUMAN. (A) Expression. Full-length G6PD protein is present in the oocyte nucleus and cytoplasm. Proteins were resolved by SDS-PAGE and G6PD was detected by Western blotting using mouse monoclonal antibody # sc-373886 (Santa Cruz Biotechnology). Migration of the 50 kDa molecular weight marker is indicated at the left. Antibody dilutions: primary 1:2000 and secondary 1:8000. (B) Activity. Glucose-6-phosphate dehydrogenase activity is detectable in homogenate of whole cytoplasms and nuclei. Final substrate concentrations: 1 mM glucose 6-phosphate, NADP^+^ as shown. The product detected is NADPH.
(PDF)

S6 Fig 6-Phosphogluconate dehydrogenase (PGD) in nuclear and cytoplasmic homogenates of *X*. *laevis* oocytes. Human UniProt link in Kirli et al., 2015: P52209 · 6PGD_HUMAN. (A) Expression. Full-length PGD protein is present in the oocyte nucleus and cytoplasm. Proteins were resolved by SDS-PAGE and PGD was detected by Western blotting using mouse monoclonal antibody # sc-398977 (Santa Cruz Biotechnology). Migration of the 50 kDa molecular weight marker is indicated at the left. Antibody dilutions: primary 1:2000 and secondary 1:8000. (B) Activity. Phosphogluconate dehydrogenase activity is detectable in homogenate of whole cytoplasms and nuclei. Final substrate concentrations: 0.5 mM 6-phosphogluconic acid, trisodium salt; 1 mM NADP^+^. The product detected is NADPH. (C) Activity–dependence of the phosphogluconate dehydrogenase reaction in nuclear homogenate on the concentration of added NADP^+^.
(PDF)

S7 Fig Individual nuclei contain the active forms of three enzymes that interconvert NADP^+^ and NADPH. (A) Summary of method. (B) Activity. Each of three individual nuclei supports the activity of IDH1, G6PD and PGD. The nuclei were from oocytes of the same ovary. Assays as in S4–S6 Figs.
(PDF)

S8 Fig Enolase α (ENO1) in nuclear and cytoplasmic homogenates of *X*. *laevis* oocytes. Human UniProt link in Kirli et al., 2015: P06733 · ENOA_HUMAN. (A) Expression. Full-length ENO1 protein is present in the oocyte nucleus and cytoplasm. Proteins were resolved by SDS-PAGE and ENO1 was detected by Western blotting using mouse monoclonal antibody # sc-271384 (Santa Cruz Biotechnology). Migration of the 50 kDa molecular weight marker is indicated at the left. Antibody dilutions: primary 1:200 and secondary 1:8000. (B) Activity. Enolase activity in homogenate of whole cytoplasms and the low-speed supernatant of nuclear homogenate. Activity in samples from oil-dissected oocytes was assessed using a colorimetric assay kit (Sigma # MAK178). In this detection system, phosphoenolpyruvate synthesis is enzymatically coupled to the production of resofurin, which has an absorbance peak of 570 nm. The reactions were performed without dithiothreitol because this reagent prevents resofurin production. Nuclear supernatant was prepared as described for Fig 5C.
(PDF)

S9 Fig Pyruvate kinase M2 (PKM2) in nuclear and cytoplasmic homogenates of *X*. *laevis* oocytes. Human UniProt link in Kirli et al., 2015: P14618 · KPYM_HUMAN. (A) Expression. Full-length PKM2 protein is present in the oocyte nucleus and cytoplasm. Proteins were resolved by SDS-PAGE and PKM2 was detected by Western blotting using rabbit polyclonal antibody # ab137791 (Abcam). Monomeric PKM2 has an expected molecular weight of 57309 Da. Migration of the 75 kDa molecular weight marker is indicated on the right. Antibody dilutions: primary 1:2000 and secondary 1:8000. (B) Oligomerization state. PKM2 in the oocyte nucleus exists mainly as a homotetramer. PKM2 protein in nuclear homogenate was resolved by native PAGE, then detected as in A. Migration of Precision Plus molecular weight markers (Bio-Rad) is on the right. PKM2 forms a homotetramer with high pyruvate kinase activity and a dimer without this activity (Gao et al., 2013). In X. laevis the predicted molecular weights of the dimer and tetramer are 114618 and 229239 Da respectively. The band pattern revealed here is consistent with high nuclear expression of tetrameric, metabolically active PKM2. This interpretation is supported by the results in (C). Native PAGE was performed as in Sen et al. (2016). The 2x loading buffer contained 200 mM KCl. Antibody dilutions: primary 1:2000, secondary 1:5000. (C) Activity. Pyruvate kinase activity in homogenates of whole cytoplasms and nuclei (Dworkin et al. (1987) previously characterized whole cell activity). In this enzyme-coupled assay pyruvate synthesized by PKM2 is used by lactate dehydrogenase in a reaction that consumes added NADH (NADH + pyruvate → lactate + NAD^+^). The coupled reaction driven by PKM2 in the presence of added phosphoenolpyruvate and ADP is supported by LDHA/B resident in the oocyte cytoplasm and nucleus (dashed traces) and is stimulated by addition of exogenous purified LDH (solid traces). Final substrate concentrations: 1.5 mM phosphoenolpyruvate, 2 mM ADP, 0.2 mM NADH. Exogenous LDH is Type III from bovine heart (Sigma # L2625). It was used at 0.4 U/40 μL reaction. Gao X, Wang H, Yang JJ, Chen J, Jie J, Li L, Zhang Y, Liu ZR. Reciprocal regulation of protein kinase and pyruvate kinase activities of pyruvate kinase M2 by growth signals. *J Biol Chem*. 2013;288:15971–15979. Sen S, Deshmane SL, Kaminski R, Amini S, Datta PK. Non-Metabolic Role of PKM2 in Regulation of the HIV-1 LTR. *J Cell Physiol*. 2017;232:517–525. Dworkin MB, Segil N, Dworkin-Rastl E. Pyruvate kinase isozymes in oocytes and embryos from the frog Xenopus laevis. *Comp Biochem Physiol B*. 1987;88:743–749.
(PDF)

S10 Fig Lactate dehydrogenase A and B chain (LDHA, LDHB) in nuclear and cytoplasmic homogenates of *X*. *laevis* oocytes. LDHA and LDHB are expressed in both the nucleus and cytoplasm, with LDHB predominating (Kirli et al., 2015). Human UniProt links in Kirli et al., 2015: P00338 · LDHA_HUMAN, P07195 · LDHB_HUMAN. (A) Activity–pH dependence. Whole oocyte LDH has been characterized previously (Claycomb and Villee, 1971). Enzyme activity is optimal at pH 10 (Nielands, 1955; Vanderlinde, 1985). In the present study whole cytoplasmic and nuclear homogenate was assayed in standard HR buffer at pH 7.4 (10 mM potassium phosphate) and HR buffer at pH 10 (75.2 mM glycine-NaOH). Activity was observed in samples of both compartments at pH 7.4 and 10, and is clearly higher at pH 10 in both compartments. Final substrate concentrations: 50 mM L-lactate (lithium salt), 5 mM NAD^+^. (B) Activity–dependence on homogenate amount at pH 10. Claycomb WC, Villee CA. Lactate dehydrogenase isozymes of Xenopus laevis: factors affecting their appearance during early development. *Dev Biol*. 1971;24:413–427. Neilands JB. Lactic dehydrogenase of heart muscle. *Meth Enzymol*. 1955;1:449–454. Vanderlinde RE. Measurement of total lactate dehydrogenase activity. *Annals Clin Lab Sci*. 1985;15:13–31.
(PDF)

S11 Fig Raw images. (PDF)

S1 Table LC-MS data for Figs 1F and 3A. *Data for cytosol previously reported in: Gill GS, Schultz MC. Multienzyme activity profiling for evaluation of cell-to-cell variability of metabolic state. *FASEB BioAdv*. 2022;4:709–723. **Nuclear expression confirmed by Western blotting–see S8, S9A and S9B Figs.
(PDF)

S2 Table The values of kinetic parameters for GAPDH in matched nuclear and cytoplasmic homogenates of Animal 3. Parameters were estimated by the Lineweaver-Burke method (four left-most columns). The values for replicates of nuclear homogenate are more divergent from one-another than are the replicates of cytoplasmic homogenate (the latter being almost identical; see two left-most columns). In the “Fold difference” columns the K_m_ and V_max_ rows show the value of a parameter for each nuclear replicate divided by the average of that parameter for the cytoplasmic replicates. For example, the K_m_ fold difference value is 4.2030/0.8793 = 4.72 for replicate 1 and 19.8651/0.8793 = 22.59 for replicate 2. If the variability between replicate samples of nuclear extract was low, then the fold difference for replicates 1 and 2 would be similar (as for K_m_ and V_max_ of the cytoplasmic enzyme). The units of K_m_ and V_max_ are mM and ΔA_340_/min respectively.
(PDF)

S3 Table Summary of S3–S10 Figs. The aim of these experiments was to explore the potential of the oocyte as a model system for broadly investigating the activity behavior of metabolic enzymes that have a pool in the cytoplasm and nucleus. Except where noted, the methods used were similar or identical to those described for GAPDH in the main text. The enzymes studied were as tabulated.
(PDF)
