# Supplementary figures and images for: Biochemical evidence that the whole compartment activity behavior of GAPDH differs between the cytoplasm and nucleus
Source: PLoS One. 2023 Aug 31;18(8):e0290892. doi: 10.1371/journal.pone.0290892 (PMC10470895; doi:10.1371/journal.pone.0290892)

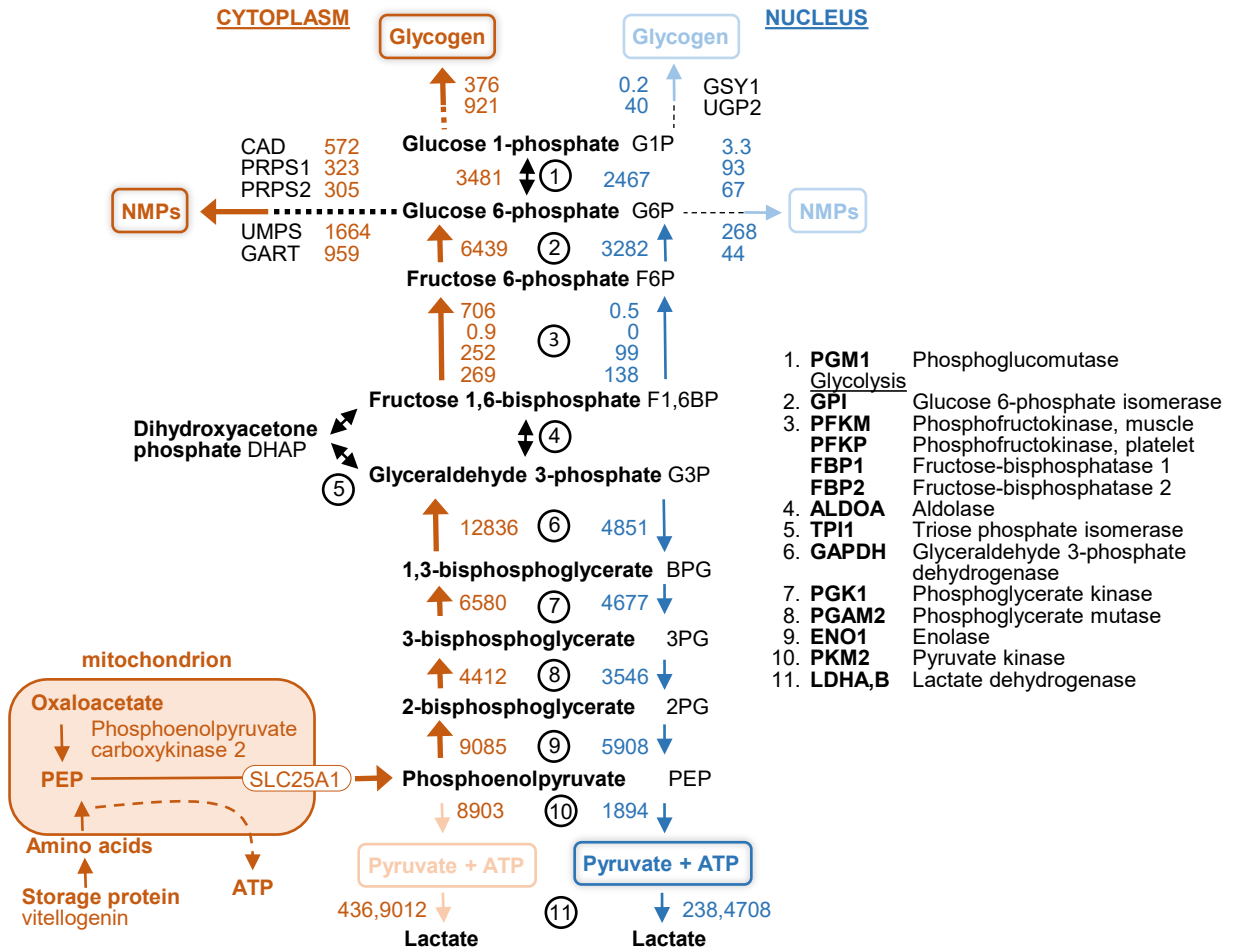

Supplement: S1 Fig — Flux through glycolytic enzymes (circled steps 2–11) occurs in opposite directions in the cytoplasm and nucleus (orange and blue arrows respectively). GAPDH acts at step 6. Relative flux intensity is indicated by line width. Dotted lines are pathways for which some steps have been omitted. Lines with black arrows identify reactions that are expected to operate similarly in the cytoplasm and nucleus. The orange and blue numbers are enzyme concentrations in nM (Kirli et al., 2015). In the cytoplasm, glycogen and nucleoside synthesis (orange boxes, dark outline) predominate over pyruvate synthesis. In the nucleus, flux through GAPDH supports operation of the payoff phase of glycolysis culminating in ATP and pyruvate synthesis (blue box, dark outline). Flux in the opposite direction is limited by low nuclear expression of critical enzymes of glycogen and nucleoside synthesis. The enzymes of glycogen synthesis shown are glycogen synthase 1 (GSY1) and UDP-glucose pyrophosphorylase (UGP2). The enzymes of nucleoside monophosphate (NMP) synthesis shown are: Carbamoyl-phosphate synthetase 2, Aspartate transcarbamylase, Dihydroorotase (CAD); Phosphoribosyl pyrophosphate synthetases 1 and 2 (PRPS1, PRPS2); Uridine monophosphate synthetase (UMPS); Phosphoribosylglycinamide formyltransferase and synthetase, Phosphoribosyl-aminoimidazole synthetase (GART). Glucose input to G6P is not shown. Most ATP production in the oocyte is fueled by mitochondrial metabolism of amino acids derived from vitellogenin. Kırlı K, Karaca S, Dehne HJ, et al. A deep proteomics perspective on CRM1-mediated nuclear export and nucleocytoplasmic partitioning. eLife. 2015;4:e11466. (PDF) [file pone.0290892.s001.pdf]

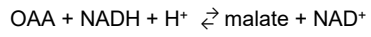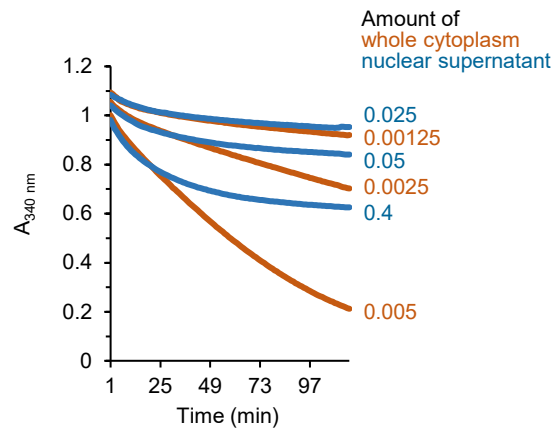

Supplement: S3 Fig — Human UniProt link in Kirli et al., 2015: P40925 · MDHC_HUMAN. Malate dehydrogenase activity in homogenates of whole cytoplasms and whole nuclei (whole cell activity was previously characterized by Gill and Schultz (2022), who also reported on the expression of full-length MDH1 in these compartments). In the present study enzyme activity was analyzed as described for whole oocyte homogenates by Gill and Schultz (2022). Gill GS, Schultz MC. Multienzyme activity profiling for evaluation of cell-to-cell variability of metabolic state. FASEB BioAdv. 2022;4:709–723. (PDF) [file pone.0290892.s003.pdf]

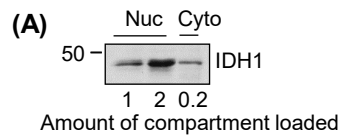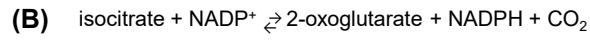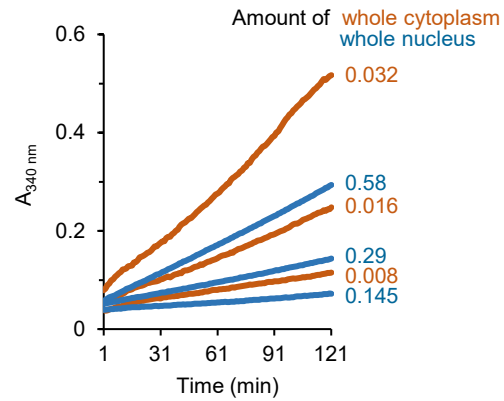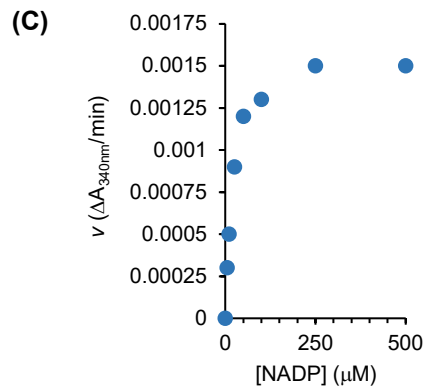

Supplement: S4 Fig — Human UniProt link in Kirli et al., 2015: O75874 · IDHC_HUMAN. (A) Expression. Full-length IDH1 protein is present in the oocyte nucleus and cytoplasm. Proteins were resolved by SDS-PAGE and IDH1 was detected by Western blotting using rabbit polyclonal antibody # ARP54787_P050 (Aviva Systems Biology). Migration of the 50 kDa molecular weight marker is indicated at the left. Antibody dilutions: primary 1:2000 and secondary 1:4000. (B) Activity. Isocitrate dehydrogenase activity is detectable in homogenate of whole cytoplasms and nuclei. Assay performed according to standard method for GAPDH. Final substrate concentrations: 2 mM isocitric acid, 1 mM NADP+. The product detected is NADPH. (C) Activity: dependence on NADP+ concentration. Dependence of the velocity of the isocitrate dehydrogenase reaction in nuclear homogenate on the concentration of added NADP+. Each reaction contained the amount of homogenate equivalent to one nucleus. v was estimated from the 5–18 min data points of progress curves. (PDF) [file pone.0290892.s004.pdf]

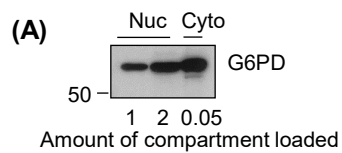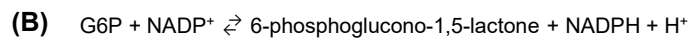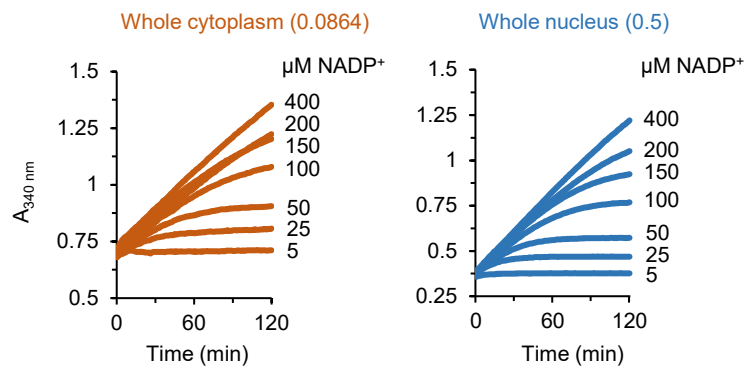

Supplement: S5 Fig — Human UniProt link in Kirli et al., 2015: P11413 · G6PD_HUMAN. (A) Expression. Full-length G6PD protein is present in the oocyte nucleus and cytoplasm. Proteins were resolved by SDS-PAGE and G6PD was detected by Western blotting using mouse monoclonal antibody # sc-373886 (Santa Cruz Biotechnology). Migration of the 50 kDa molecular weight marker is indicated at the left. Antibody dilutions: primary 1:2000 and secondary 1:8000. (B) Activity. Glucose-6-phosphate dehydrogenase activity is detectable in homogenate of whole cytoplasms and nuclei. Final substrate concentrations: 1 mM glucose 6-phosphate, NADP+ as shown. The product detected is NADPH. (PDF) [file pone.0290892.s005.pdf]

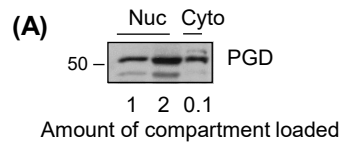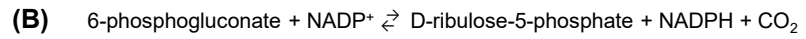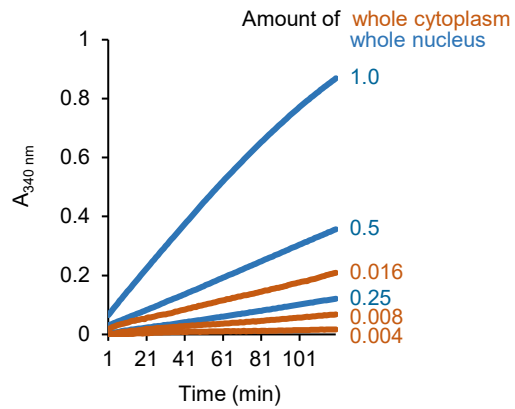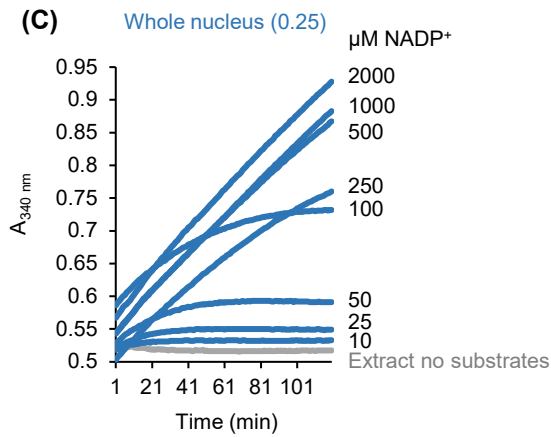

Supplement: S6 Fig — Human UniProt link in Kirli et al., 2015: P52209 · 6PGD_HUMAN. (A) Expression. Full-length PGD protein is present in the oocyte nucleus and cytoplasm. Proteins were resolved by SDS-PAGE and PGD was detected by Western blotting using mouse monoclonal antibody # sc-398977 (Santa Cruz Biotechnology). Migration of the 50 kDa molecular weight marker is indicated at the left. Antibody dilutions: primary 1:2000 and secondary 1:8000. (B) Activity. Phosphogluconate dehydrogenase activity is detectable in homogenate of whole cytoplasms and nuclei. Final substrate concentrations: 0.5 mM 6-phosphogluconic acid, trisodium salt; 1 mM NADP+. The product detected is NADPH. (C) Activity–dependence of the phosphogluconate dehydrogenase reaction in nuclear homogenate on the concentration of added NADP+. (PDF) [file pone.0290892.s006.pdf]

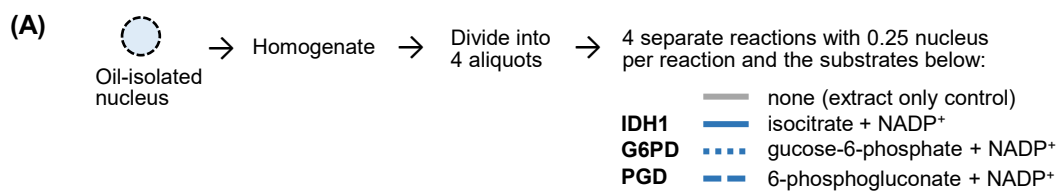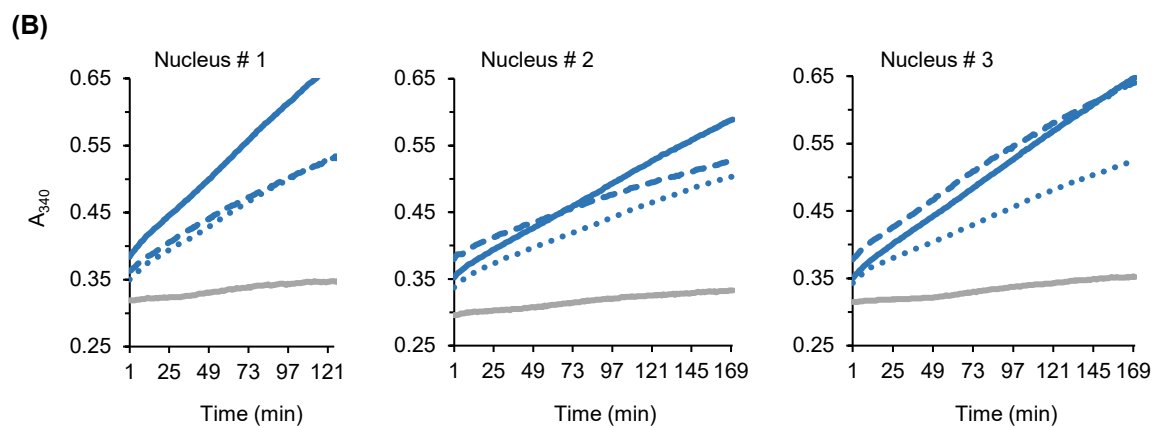

Supplement: S7 Fig — (A) Summary of method. (B) Activity. Each of three individual nuclei supports the activity of IDH1, G6PD and PGD. The nuclei were from oocytes of the same ovary. Assays as in S4–S6 Figs. (PDF) [file pone.0290892.s007.pdf]

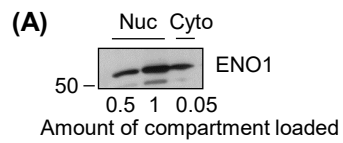

**(B)** 2-phospho-D-glycerate  $\rightleftharpoons$  phosphoenolpyruvate + H<sub>2</sub>O

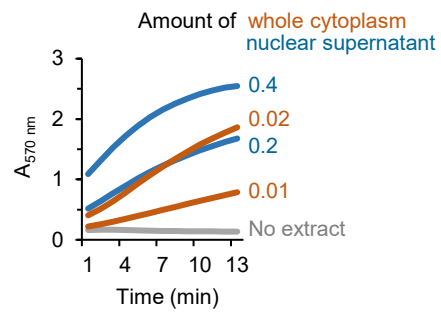

Supplement: S8 Fig — Human UniProt link in Kirli et al., 2015: P06733 · ENOA_HUMAN. (A) Expression. Full-length ENO1 protein is present in the oocyte nucleus and cytoplasm. Proteins were resolved by SDS-PAGE and ENO1 was detected by Western blotting using mouse monoclonal antibody # sc-271384 (Santa Cruz Biotechnology). Migration of the 50 kDa molecular weight marker is indicated at the left. Antibody dilutions: primary 1:200 and secondary 1:8000. (B) Activity. Enolase activity in homogenate of whole cytoplasms and the low-speed supernatant of nuclear homogenate. Activity in samples from oil-dissected oocytes was assessed using a colorimetric assay kit (Sigma # MAK178). In this detection system, phosphoenolpyruvate synthesis is enzymatically coupled to the production of resofurin, which has an absorbance peak of 570 nm. The reactions were performed without dithiothreitol because this reagent prevents resofurin production. Nuclear supernatant was prepared as described for Fig 5C. (PDF) [file pone.0290892.s008.pdf]

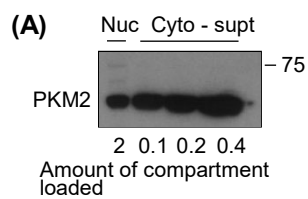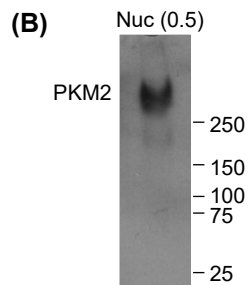

**(C)** phosphoenolpyruvate + ADP  $\rightleftharpoons$  pyruvate + ATP

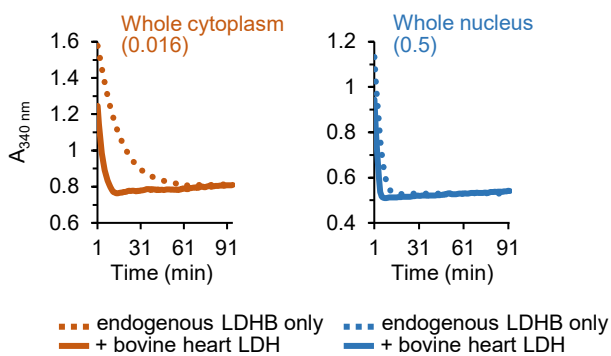

Supplement: S9 Fig — Human UniProt link in Kirli et al., 2015: P14618 · KPYM_HUMAN. (A) Expression. Full-length PKM2 protein is present in the oocyte nucleus and cytoplasm. Proteins were resolved by SDS-PAGE and PKM2 was detected by Western blotting using rabbit polyclonal antibody # ab137791 (Abcam). Monomeric PKM2 has an expected molecular weight of 57309 Da. Migration of the 75 kDa molecular weight marker is indicated on the right. Antibody dilutions: primary 1:2000 and secondary 1:8000. (B) Oligomerization state. PKM2 in the oocyte nucleus exists mainly as a homotetramer. PKM2 protein in nuclear homogenate was resolved by native PAGE, then detected as in A. Migration of Precision Plus molecular weight markers (Bio-Rad) is on the right. PKM2 forms a homotetramer with high pyruvate kinase activity and a dimer without this activity (Gao et al., 2013). In X. laevis the predicted molecular weights of the dimer and tetramer are 114618 and 229239 Da respectively. The band pattern revealed here is consistent with high nuclear expression of tetrameric, metabolically active PKM2. This interpretation is supported by the results in (C). Native PAGE was performed as in Sen et al. (2016). The 2x loading buffer contained 200 mM KCl. Antibody dilutions: primary 1:2000, secondary 1:5000. (C) Activity. Pyruvate kinase activity in homogenates of whole cytoplasms and nuclei (Dworkin et al. (1987) previously characterized whole cell activity). In this enzyme-coupled assay pyruvate synthesized by PKM2 is used by lactate dehydrogenase in a reaction that consumes added NADH (NADH + pyruvate → lactate + NAD+). The coupled reaction driven by PKM2 in the presence of added phosphoenolpyruvate and ADP is supported by LDHA/B resident in the oocyte cytoplasm and nucleus (dashed traces) and is stimulated by addition of exogenous purified LDH (solid traces). Final substrate concentrations: 1.5 mM phosphoenolpyruvate, 2 mM ADP, 0.2 mM NADH. Exogenous LDH is Type III from bovine heart (Sigma # L2625). It was used at [file pone.0290892.s009.pdf]

**(A)** lactate + NAD<sup>+</sup>  $\rightleftharpoons$  pyruvate + NADH + H<sup>+</sup>

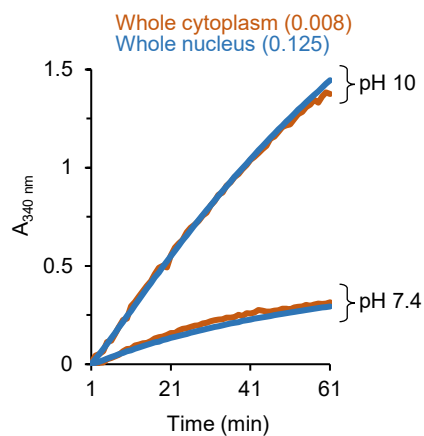

**(B)**

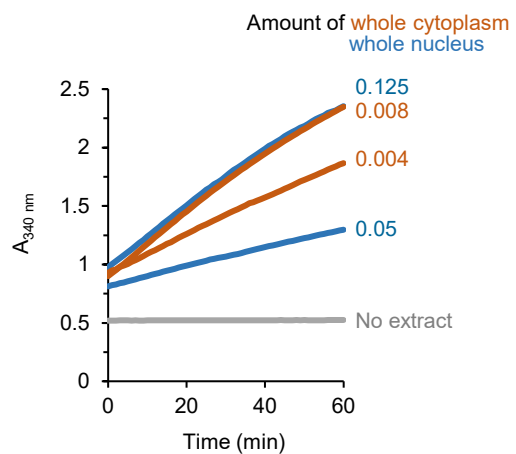

Supplement: S10 Fig — LDHA and LDHB are expressed in both the nucleus and cytoplasm, with LDHB predominating (Kirli et al., 2015). Human UniProt links in Kirli et al., 2015: P00338 · LDHA_HUMAN, P07195 · LDHB_HUMAN. (A) Activity–pH dependence. Whole oocyte LDH has been characterized previously (Claycomb and Villee, 1971). Enzyme activity is optimal at pH 10 (Nielands, 1955; Vanderlinde, 1985). In the present study whole cytoplasmic and nuclear homogenate was assayed in standard HR buffer at pH 7.4 (10 mM potassium phosphate) and HR buffer at pH 10 (75.2 mM glycine-NaOH). Activity was observed in samples of both compartments at pH 7.4 and 10, and is clearly higher at pH 10 in both compartments. Final substrate concentrations: 50 mM L-lactate (lithium salt), 5 mM NAD+. (B) Activity–dependence on homogenate amount at pH 10. Claycomb WC, Villee CA. Lactate dehydrogenase isozymes of Xenopus laevis: factors affecting their appearance during early development. Dev Biol. 1971;24:413–427. Neilands JB. Lactic dehydrogenase of heart muscle. Meth Enzymol. 1955;1:449–454. Vanderlinde RE. Measurement of total lactate dehydrogenase activity. Annals Clin Lab Sci. 1985;15:13–31. (PDF) [file pone.0290892.s010.pdf]
