# Supplementary material for: Biochemical evidence that the whole compartment activity behavior of GAPDH differs between the cytoplasm and nucleus
Source: PLoS One. 2023 Aug 31;18(8):e0290892. doi: 10.1371/journal.pone.0290892 (PMC10470895; doi:10.1371/journal.pone.0290892)
Supplement: S2 Fig — A challenge in biochemical studies of nuclear enzymes is that isolated nuclei can be associated with cytoplasmic material. This material can be in the form of cytoplasm adhering to the nuclear exterior and cytoplasm present in invaginations of the nuclear envelope (which form a “nucleoplasmic reticulum”; Drozdz and Vaux, 2017). To our knowledge the amount of such cytoplasmic material associated with nuclei isolated from tissue culture cells has not been estimated. Such an estimate can however be made for the oil-isolated X. laevis oocyte (in the diagram the nucleoplasmic reticulum has been enlarged for clarity). This estimate is based on the known volumes of the nucleus and cytosol of stage VI oocytes (Gurdon and Wickens, 1983), and images in two studies of the X. laevis oocyte. The first reveals the nucleoplasmic reticulum in an equatorial section of a stage VI oocyte (plate 1 in Hausen and Riebesell, 1991; link available at https://www.xenbase.org/entry/doNewsRead.do?id=613). The section was stained with a classical dye that colors the cytosol blue. Penetrations of cytoplasm into the nucleus are evident. Using a high-resolution download of this image, we employed the “point hit” method (Elias and Hyde, 1983) to estimate the volume of the nuclear interior that is cytoplasm. That volume is 1.22 nL. The second image is a transmission electron micrograph showing the rim of cytoplasmic material of an oil-isolated nucleus (Paine et al., 1992). From this image we can estimate the maximum depth of the cytoplasmic rim (0.5 mm) and the corresponding volume of the cytoplasmic rim (0.28 nL). The volume of cytoplasm contributed to oil isolated nuclei by the nucleoplasmic reticulum and cytoplasmic rim is therefore approximately 1.5 nL. Based on this estimate and the concentration of GAPDH reported for oocyte cytoplasm (Kirli et al. (2015), the nuclear concentration of GAPDH would be 4.8135 nM if the only source of the GAPDH was nucleus-associated cytoplasm. The actual nuclear c [file pone.0290892.s002.pdf]

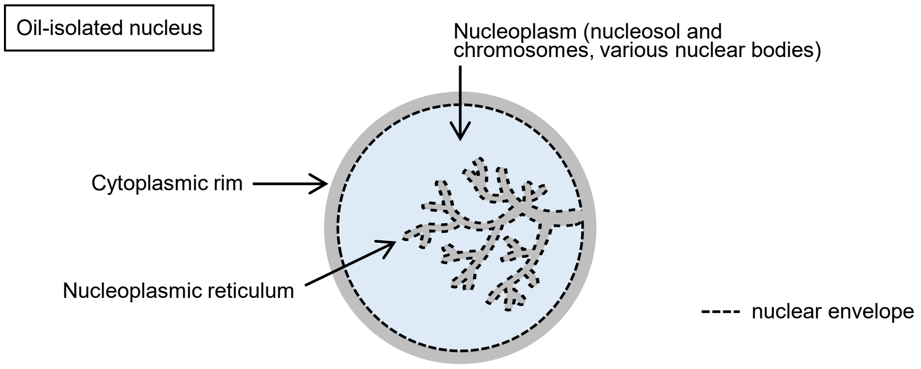
Oil-isolated nucleus
Nucleoplasm (nucleosol and chromosomes, various nuclear bodies)
Cytoplasmic rim
Nucleoplasmic reticulum
nuclear envelope
