# Supplementary material for: Biochemical evidence that the whole compartment activity behavior of GAPDH differs between the cytoplasm and nucleus
Source: PLoS One. 2023 Aug 31;18(8):e0290892. doi: 10.1371/journal.pone.0290892 (PMC10470895; doi:10.1371/journal.pone.0290892)
Supplement: S11 Fig — (PDF) [file pone.0290892.s011.pdf]

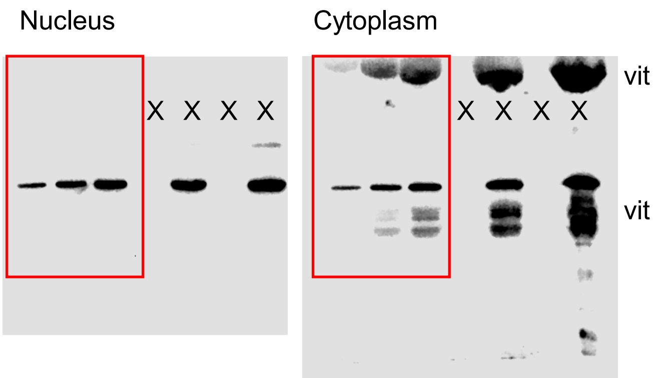

Figure 1C ECL/film

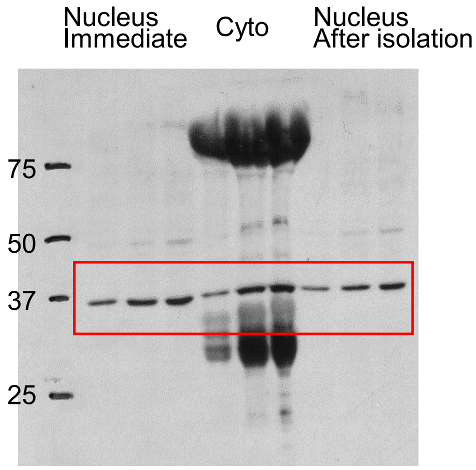

Figure 1D ECL/filmTIF

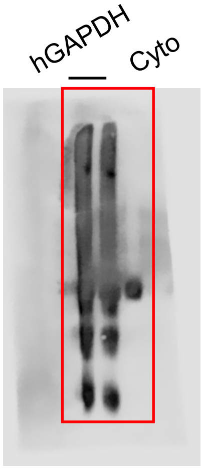

Figure 1E left panel TIF
ECL/LI-COR

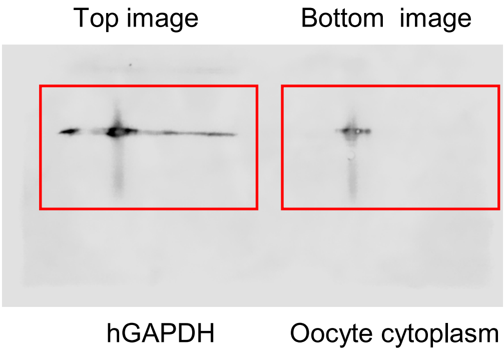

Figure 1E middle panel PNG
ECL/LI-COR

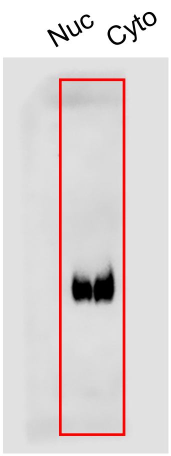

Figure 1E right panel TIF
ECL/LI-COR

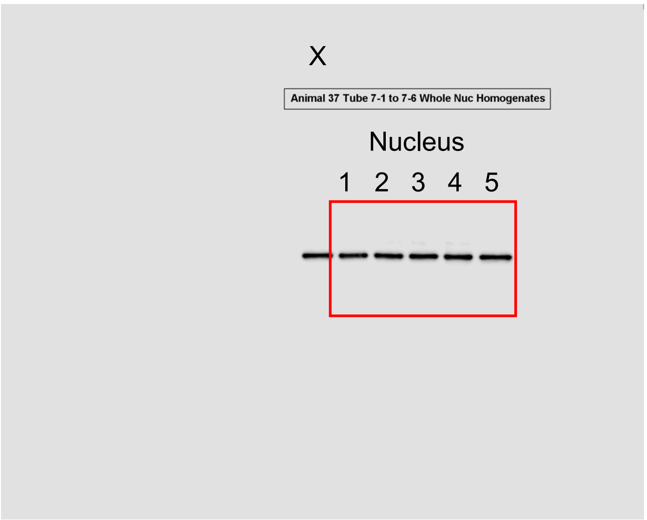

Figure 3B PNG
ECL/LI-COR

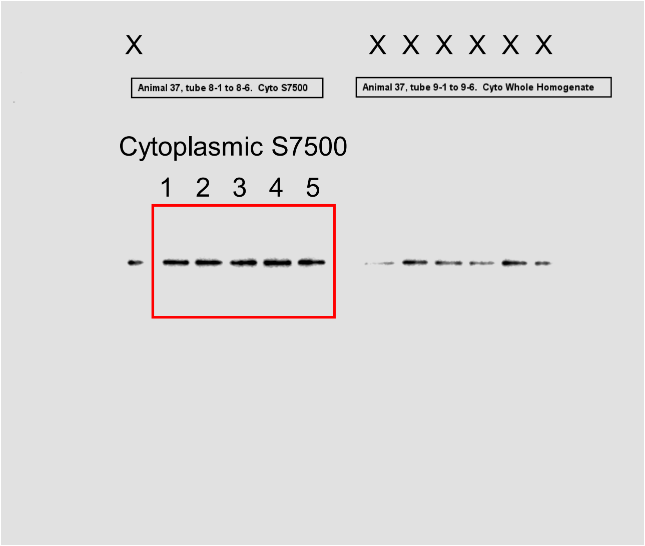

Figure 3C PNG
ECL/LI-COR

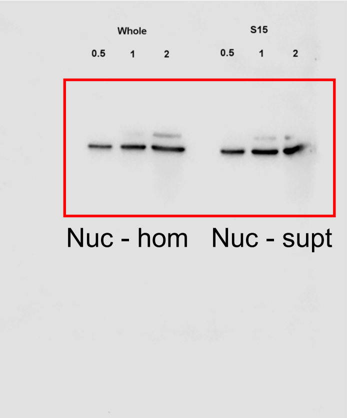

Figure 5C, top panel
ECL/film TIF

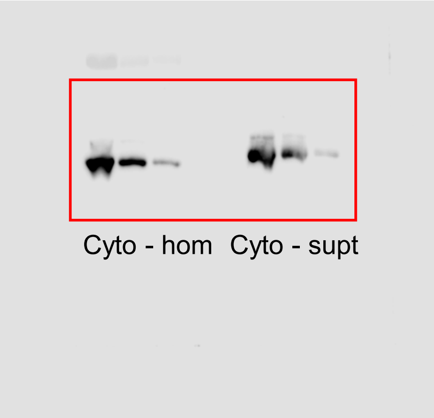

Figure 5C, middle panel
ECL/film PNG

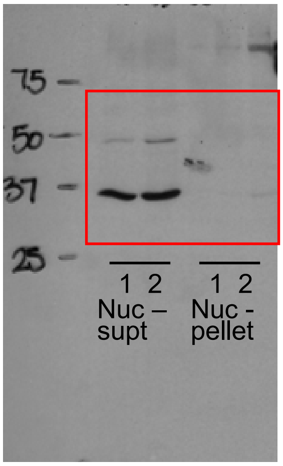

Figure 5C, bottom panel
ECL/film PNG

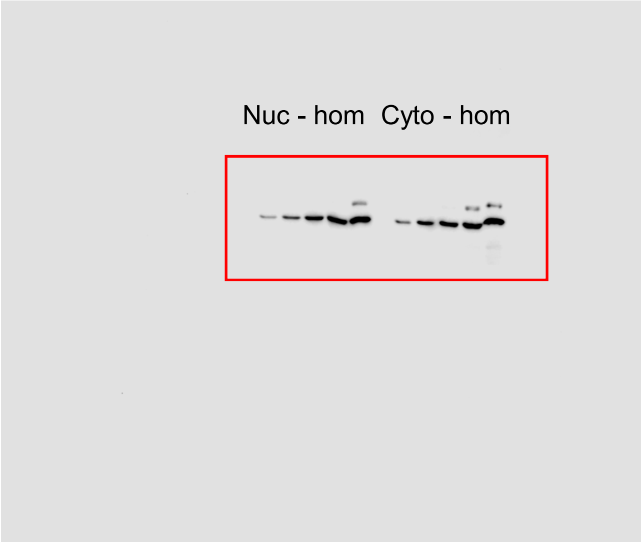

Figure 5E, middle panel ECL/LI-COR TIF
