# Supplementary material for: Biochemical evidence that the whole compartment activity behavior of GAPDH differs between the cytoplasm and nucleus
Source: PLoS One. 2023 Aug 31;18(8):e0290892. doi: 10.1371/journal.pone.0290892 (PMC10470895; doi:10.1371/journal.pone.0290892)
Supplement: S1 Table — *Data for cytosol previously reported in: Gill GS, Schultz MC. Multienzyme activity profiling for evaluation of cell-to-cell variability of metabolic state. FASEB BioAdv. 2022;4:709–723. **Nuclear expression confirmed by Western blotting–see S8, S9A and S9B Figs. (PDF) [file pone.0290892.s012.pdf]

		Nucleus		Cytoplasm	
**Protein**	**Sample**	**% of total # PSMs**	**% rank**	**% of total # PSMs**	**% rank**
NPM2	1	2.317779811	0.9757	0	
	2	1.900882536	0.9625	0	
	3	0.624311421	0.862	0	
	4	0.6875	0.8643	0	
	5	2.826420891	0.9839	0	
	6	2.048247585	0.9671	0	
ATP6V1B2	1	0		0.157260177	0.5497
	2	0		0.1594642	0.5601
	3	0		0.16523463	0.5302
	4	0		0.270075615	0.7158
	5	0		0.128534697	0.5305
	6	0		0.16339869	0.5858
GAPDH*	1	1.219884111	0.9109	2.201642478	0.9917
	2	1.391717571	0.9333	1.70626694	0.9781
	3	1.505692251	0.9348	0.82617315	0.9221
	4	1.78125	0.9418	1.602448649	0.9808
	5	0.983102919	0.9116	2.027991886	0.9902
	6	1.275626424	0.9061	2.058823494	0.9924
GPI	1	1.006404392	0.9028	1.590075123	0.9723
	2	0.882552606	0.8875	1.80194546	0.9808
	3	0.918105031	0.9157	2.247190968	0.9884
	4	0.53125	0.8333	1.728483936	0.9863
	5	1.505376344	0.9317	1.828049024	0.9853
	6	1.274465164	0.9061	1.699346376	0.9873
PFKM	1	0		0.069893412	0.2182
	2	0		0.06378568	0.1748
	3	0		0.049570389	0.1181
	4	0		0.036010082	0.0027
	5	0		0.042844899	0.1246
	6	0		0.049019607	0.1363
ALDOC	1	0.579444953	0.838	0.856194297	0.9309
	2	0.475220634	0.8208	1.30760644	0.9699
	3	0.29379361	0.7203	0.214805019	0.6167
	4	0.3125	0.7364	0.954267173	0.9398
	5	0.64516129	0.8514	1.242502071	0.9682
	6	0.819299034	0.8403	0.931372533	0.9444
TPI	1	3.537663922	0.9878	1.502708358	0.9668
	2	3.190767114	0.9833	1.40328496	0.9754
	3	7.381564451	0.9961	1.586252448	0.9798
	4	5	0.9922	1.494418403	0.9726
	5	2.91858679	0.9879	1.670951061	0.9828
	6	2.548930328	0.9765	1.323529389	0.9671
PGK1	1	0.426959439	0.7854	0.436833825	0.837
	2	0.644942289	0.8541	0.44649976	0.8415
	3	0.514138817	0.816	0.594844668	0.8703
	4	0.59375	0.8488	0.378105861	0.8142
	5	0.337941628	0.7349	0.528420421	0.8801
	6	0.455580866	0.75	0.375816987	0.8333
PGAM1	1	0.152485514	0.4817	0.192206883	0.5911
	2	0.135777324	0.3458	0.27108914	0.724
	3	0.220345207	0.6245	0.264375408	0.7002
	4	0.21875	0.6511	0.162045369	0.5573
	5	0.122887865	0.3654	0.242787761	0.731
	6	0.091033226	0.0046	0.196078428	0.6464
ENO1**	1	0.9959	0.57944495	4.560545133	0.9972
	2	0.9958	0.37338764	5.61313984	0.9972
	3	0.9923	0.55086302	5.188367382	0.9971
	4	0.9883	0.28125	5.76161312	0.9972
	5	0.9919	0.64516129	4.641530725	0.9975
	6	0.9906	1.13791533	4.068627381	0.9974
PKM2**	1	0.579444953	0.838	1.764808653	0.9806
	2	0.373387641	0.75	2.24844522	0.989
	3	0.550863019	0.8275	2.461995987	0.9913
	4	0.28125	0.7131	2.412675494	0.9945
	5	0.64516129	0.8514	2.113681684	0.9926
	6	1.137915325	0.8873	1.977124149	0.9898
LDHB	1	1.829826167	0.9554	1.030927827	0.9447
	2	1.900882536	0.9625	0.98867804	0.9508
	3	4.039662137	0.9885	0.875743539	0.9365
	4	2.625	0.9728	1.116312542	0.959
	5	1.689708141	0.9598	0.99971431	0.9511
	6	1.957214359	0.953	1.062091485	0.957
