# Supplementary material for: Biochemical evidence that the whole compartment activity behavior of GAPDH differs between the cytoplasm and nucleus
Source: PLoS One. 2023 Aug 31;18(8):e0290892. doi: 10.1371/journal.pone.0290892 (PMC10470895; doi:10.1371/journal.pone.0290892)
Supplement: S2 Table — Parameters were estimated by the Lineweaver-Burke method (four left-most columns). The values for replicates of nuclear homogenate are more divergent from one-another than are the replicates of cytoplasmic homogenate (the latter being almost identical; see two left-most columns). In the “Fold difference” columns the Km and Vmax rows show the value of a parameter for each nuclear replicate divided by the average of that parameter for the cytoplasmic replicates. For example, the Km fold difference value is 4.2030/0.8793 = 4.72 for replicate 1 and 19.8651/0.8793 = 22.59 for replicate 2. If the variability between replicate samples of nuclear extract was low, then the fold difference for replicates 1 and 2 would be similar (as for Km and Vmax of the cytoplasmic enzyme). The units of Km and Vmax are mM and ΔA340/min respectively. (PDF) [file pone.0290892.s013.pdf]

Animal 3

Replicate	Cytoplasm 1	Cytoplasm 2	Nucleus 1	Nucleus 2	Fold difference - nuc replicate ÷ cyto average 1	Fold difference - nuc replicate ÷ cyto average 2
K_m	0.8692	0.8893	4.2030	19.8651	4.72	22.59
avg.		*0.8793*		*12.0341*		
V_{max}	0.0178	0.0195	0.0603	0.1804	3.22	9.65
avg.		*0.0187*		*0.1204*		
