# Supplementary material for: Biochemical evidence that the whole compartment activity behavior of GAPDH differs between the cytoplasm and nucleus
Source: PLoS One. 2023 Aug 31;18(8):e0290892. doi: 10.1371/journal.pone.0290892 (PMC10470895; doi:10.1371/journal.pone.0290892)
Supplement: S3 Table — The aim of these experiments was to explore the potential of the oocyte as a model system for broadly investigating the activity behavior of metabolic enzymes that have a pool in the cytoplasm and nucleus. Except where noted, the methods used were similar or identical to those described for GAPDH in the main text. The enzymes studied were as tabulated. (PDF) [file pone.0290892.s014.pdf]

			Concentration (nM)*		
Enzyme		Pathway	Cytoplasm	Nucleus	Figure**
Malate dehydrogenase 1	MDH1	Malate-aspartate shuttle	6509.8	4243.0	S3
Isocitrate dehydrogenase 1	IDH1	NADPH production	917.4	629.9	S4
Glucose 6-phosphate dehydrogenase	G6PD	Pentose phosphate pathway	836.4	180.6	S5
6-Phosphogluconate dehydrogenase	PGD	Pentose phosphate pathway	938.5	722.4	S6
Enolase α	ENO1	Glycolysis	9084.9	5908.4	S8
Pyruvate kinase M2	PKM2	Glycolysis	8902.7	1894.4	S9
Lactate dehydrogenase (A, B chains)	LDHA/B	Glycolysis yields its major substrate	9448.6	4945.5	S10

*From Kirli et al., 2015.

**Supplementary Figure S7 shows that individual nuclei contain active IDH1, G6PD and PGD.
